# The Effectiveness of Virtual Reality Interventions on Smoking, Nutrition, Alcohol, Physical Activity and/or Obesity Risk Factors: A Systematic Review

**DOI:** 10.3390/ijerph191710821

**Published:** 2022-08-30

**Authors:** Peter Tatnell, Prince Atorkey, Flora Tzelepis

**Affiliations:** 1 School of Medicine and Public Health, University of Newcastle, Callaghan, NSW 2308, Australia; 2 Hunter New England Population Health, Hunter New England Local Health District, Wallsend, NSW 2287, Australia; 3 Hunter Medical Research Institute, New Lambton Heights, NSW 2305, Australia

**Keywords:** virtual reality, augmented reality, avatar, smoking, nutrition, alcohol, physical activity, obesity

## Abstract

To our knowledge, no systematic reviews have examined the effectiveness of virtual reality (VR) interventions across all smoking, nutrition, alcohol, physical activity, and/or obesity (SNAPO) risk factors. This systematic review assessed the effectiveness of VR interventions on reducing SNAPO risks compared to control groups or other interventions. MEDLINE, EMBASE, Scopus, PsycINFO, and CENTRAL were searched to identify eligible studies published to 7 October 2021. Two reviewers independently completed screening, data extraction and quality assessment. Twenty-six studies were included, five on smoking, twelve on physical activity (PA), six on obesity, one on PA and obesity, one on obesity and nutrition, and one on obesity, nutrition and PA. VR was effective for smoking cessation in three studies and for smoking reduction in four studies. Seven studies had significantly higher PA in the VR group, and one study found significantly higher PA in a comparator group. Two studies showed VR was more effective at reducing BMI or weight than comparators. Three multiple health risks studies showed mixed results. The remaining studies found no significant difference between VR and control/comparators. VR appears promising for the treatment of smoking, nutrition, PA, and obesity risks; however, further randomised trials are needed.

## 1. Introduction

Smoking tobacco, poor nutrition, risky alcohol use, physical inactivity, and/or obesity (SNAPO) are major preventable risk factors of chronic diseases [1,2]. Tobacco use is responsible for over 8 million deaths each year worldwide [3], and poor nutrition such as high intake of sodium, low grains and low fruit intake has been attributed to 11 million deaths globally [4]. Harmful alcohol use has been attributed to 3 million deaths annually worldwide [5] and there are 1.6 million deaths each year due to physical inactivity [1]. Globally, 8% of deaths have been linked to obesity in 2017 [6].

Virtual reality (VR), augmented reality, and technology using computer-generated avatars in a virtual environment (VE) have been used to deliver behavioural treatment, education or monitoring for various conditions [7]. VR can be categorised into three levels of immersion: fully immersive VR, semi-immersive VR and non-immersive VR [8]. Fully immersive VR utilise a three-dimensional, computer-generated environment or image that can be viewed/interacted with using wearable technology, such as a VR headset [8]. Typically, fully immersive VR requires VR glasses that utilise a stereoscopic 3D environment by splitting the display between the user’s eyes, giving the impression the user is in the virtual environment [8]. Semi-immersive VR provides immersion using sensors, powerful displays and graphics, and/or replica physical settings such as an aeroplane cockpit to interact with a virtual setting [8]. Non-immersive VR includes avatar-based programs and typically uses a computer or console system, monitor or TV display, where interactions in a VE occur via keyboard, mouse, or controller [8]. Advantages of adopting VR in a clinical or health setting include the potential for greater learning and engagement in treatments or education, it can be used remotely as it is delivered via the internet, and it allows interventions to be more flexible [7].

A number of systematic reviews have examined the effectiveness of VR interventions on improving smoking, alcohol, physical activity and/or obesity risk factors [9,10,11,12,13,14,15,16,17,18,19,20,21,22] but none have focussed on nutrition. However, these reviews focus primarily on a single health risk behaviour [9,10,11,12,13,14,15,16,17,18,19], the outcome of cravings [20,21,22], a specific type of VR such as avatar-based studies [15], or included non-randomised controlled studies [20]. In relation to smoking tobacco, systematic reviews have shown promising results showing VR cue exposure therapy can be used to reduce cravings [16,20]. However, results are mixed on whether VR cue exposure is better than current interventions such as cognitive behavioural therapy (CBT) [16,20]. The effectiveness of VR cue exposure on smoking cessation appeared promising in these reviews, however, there were very few studies and its benefit over existing strategies such as CBT was inconclusive [16,20]. Furthermore, these reviews [16,20] included studies with no control group, non-randomised studies, compared VR with VR comparators, or the review focused on the type of therapies delivered through VR and not the effectiveness of VR itself. Existing systematic reviews examining VR interventions and alcohol have focused on VR cue exposure therapy as a treatment for cravings, driving simulations, or training and not the effectiveness of VR on cessation of alcohol use/misuse [11,14,20,21,22].

Eight systematic reviews have examined the effectiveness of VR interventions on physical activity [9,10,12,13,15,17,18,19]. One review examining the use of VR in cardiac rehabilitation (CR) programs found increases in physical activity when using VR technology [13]. However, this review [13] exclusively looked at CR programs and included studies that were not RCTs, did not use control groups, and focused on outcomes such as functionality and mobility. Another review [19] found that ‘active video games’ were able to generate light to moderate PA. However, of the 13 intervention studies only three found VR interventions significantly increased PA or exercise attendance [19]. Existing systematic reviews have shown VR use for physical rehabilitation in stroke patients [12,17], and the elderly [9,10,18] is well established. Only one systematic review assessed the effectiveness of avatars in weight management and although the evidence was limited, the use of avatars to positively impact weight loss was promising [15]. 

In addition to the effectiveness of VR interventions on reducing SNAPO risk factors, other factors such as use, satisfaction/acceptability of support received, and economic costs are also important to consider. None of the existing systematic reviews [9,10,11,12,13,14,15,16,17,18,19,20,21,22] have included outcomes on use of VR, user satisfaction/acceptability or costs, although, four reviews have noted there is a need for more studies to report costs [11,16,20,21]. 

Given the limitations of the existing systematic reviews [9,10,11,12,13,14,15,16,17,18,19,20,21,22], and current gaps in the literature, a comprehensive systematic review that examines the effectiveness of VR interventions across SNAPO risk factors is needed. The primary aim of this systematic review was to examine the effectiveness of VR interventions on reducing smoking tobacco, nutrition, alcohol, physical activity and/or obesity risk factors compared to (1) no intervention or minimal support control groups or (2) other behavioural or non-behavioural interventions.

The secondary aim of this systematic review was to examine use, satisfaction/acceptability of support received and economic costs of VR interventions for SNAPO risk factors compared to control/comparator groups.

## 2. Materials and Methods

### 2.1. Design and Registration

This systematic review was registered in the International Prospective Register of Systematic Reviews (PROSPERO; Registration number: CRD42021279086) and follows the Preferred Reporting Items for Systematic Reviews and Meta-Analyses (PRISMA) [23] guidelines.

### 2.2. Search Strategy

Online databases MEDLINE, EMBASE, Scopus, PsycINFO, and Cochrane Register of Controlled Trials (CENTRAL) were searched to identify eligible studies published to 7 October 2021. The search strategy included keywords and Medical Subject Heading (MeSH) terms (See Supplementary Table S1) and was split into three categories; (1) the type of VR intervention or technology used (i.e., virtual reality, augmented reality, avatar studies); (2) SNAPO risk factors (i.e., smoking, poor nutrition, alcohol use, physical inactivity, or obesity); and (3) study design (e.g., randomized controlled trial, randomized trial).

### 2.3. Inclusion Criteria

Studies were included if they met the following inclusion criteria:

1. Study design: Randomised controlled trials, cluster randomised controlled trials, randomised trials, and cluster randomised trials.
2. Participants: People engaging in any of the following health risk factors, individually or in combination: tobacco use, inadequate nutrition, risky alcohol consumption, insufficient physical activity, and obesity; any age, any gender, from any country.
3. Setting: Any setting (e.g., community, health setting).
4. Intervention: Fully immersive, semi-immersive and non-immersive VR technology used to treat SNAPO risk factors. These types of VR immersion are defined as: fully immersive that required VR glasses that utilise a stereoscopic 3D environment by splitting the display between the user’s eyes; semi-immersive which provided immersion through the use of sensors, powerful displays, or replica physical setting such as an aeroplane cockpit to interact with a virtual setting; and non-immersive that consists of a virtual representation of the participant (e.g., avatar), displayed on a PC or console system, augmented reality, and related technology using computer-generated avatars in a VE.
5. Comparators: No intervention or minimal support control groups, other modes of behavioural or non-behavioural interventions.
6. Language: Studies published in English.
7. Outcome measures:

Smoking: Smoking cessation or reduction in tobacco use (e.g., point prevalence abstinence, prolonged abstinence, reduction in cigarettes smoked per day).

Nutrition: Serves of fruit and/or vegetables and calories.

Alcohol: Number of alcoholic drinks consumed or risky or hazardous alcohol use.

Physical activity: Number of minutes of moderate or vigorous physical activity or metabolic equivalent (MET) minutes or physical activity levels (including where peak Vo2, energy expenditure, or heart rate are measures of level of activity).

Obesity: Body Mass Index and/or weight.

### 2.4. Exclusion Criteria

Studies that used VR or computer technology that did not place the participant or virtual representation of the participant into a VE were excluded. Studies where physical activity related to fitness or functionality only (e.g., rehabilitation of limb function) were excluded.

### 2.5. Study Selection

All search results were uploaded into Endnote X9 where duplicates were removed. Records were then imported into Covidence for screening. Titles and abstracts were first screened for eligibility by two authors (PT and PA) independently. The full-text of articles where eligibility could not be determined based on the title and abstract screening were retrieved and two authors (PT and PA) independently completed full-text screening. Reasons for the exclusion of studies from full-text screening were recorded. Discrepancies were resolved through discussions between the two reviewers, and if unresolved a third team member (FT) was consulted.

### 2.6. Data Extraction

Two reviewers (PT and PA) independently extracted data from included studies using a data extraction form adapted from the Cochrane Effective Practice and Organisation of Care (EPOC) Group’s template. Any discrepancies between information extracted by the two reviewers were discussed until agreement was reached. If consensus was not met, the third team member (FT) was consulted. The following information was extracted:

- Publication details: author(s) names, year of publication, country of study and years data collected
- Setting: population based, community or clinic-based
- Study design: randomised controlled trials, cluster randomised controlled trials, randomised trials or cluster randomised trials
- Population: demographics (age, gender, level of education, employment), sample size, recruitment methods, eligibility criteria, retention rates
- Intervention: type of virtual reality intervention
- Control/Comparators: type of control (e.g., no intervention, minimal support) or comparators (e.g., in person, telephone)
- Outcomes: smoking cessation/reduction, nutrition, alcohol use, physical activity, obesity, use, satisfaction and/or acceptability of support received and economic cost.

### 2.7. Quality Assessment

The Quality Assessment Tool for Quantitative Studies developed by the Effective Public Health Practice Project was used to assess methodological quality [24]. This tool assessed the quality of a study’s methods related to selection bias, confounders, blinding, data collection methods, withdrawals and dropouts. For each study these components were rated as “strong”, “moderate” or “weak” quality. The Quality Assessment Tool for Quantitative Studies Dictionary was used to rate each of the six components [25]. An overall global rating was then assigned to each study with studies classified as ‘strong’ (no weak ratings), ‘moderate’ (one weak rating) or ‘weak’ (two or more weak ratings).

Quality assessment was conducted independently by two members of the review team (PT and PA). Differences were resolved through discussions between these two reviewers and if unresolved a third team member (FT) was consulted for adjudication if necessary.

### 2.8. Data Synthesis

This narrative synthesis firstly synthesised eligible studies based on the individual or combinations of SNAPO risk factors targeted and secondly by the types of outcomes measured. Thirdly, studies were synthesised according to whether participants were from a clinical or non-clinical population.

## 3. Results

**Figure 1:**
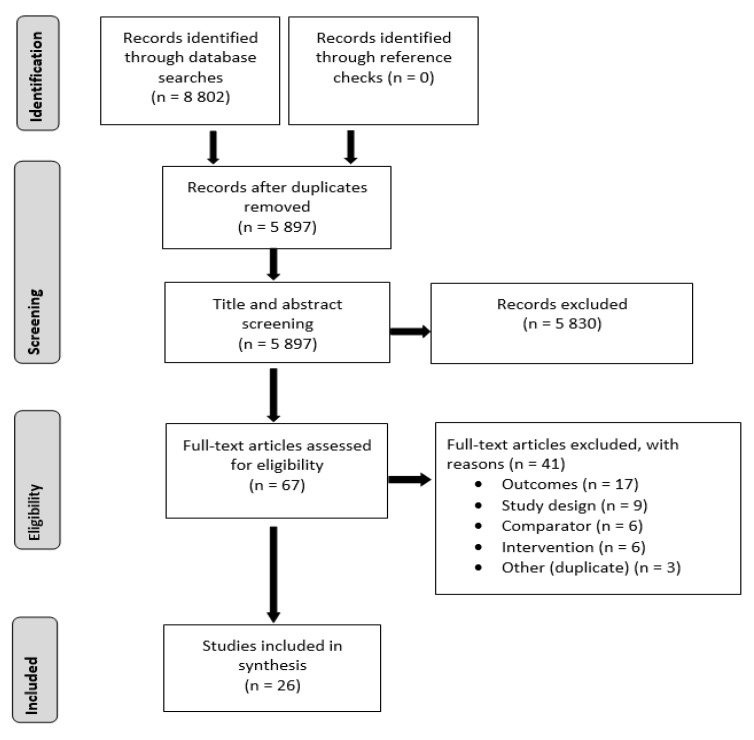
PRISMA flow chart of screening and selection process.

Database searches produced 8802 records, 5897 were retained after duplicates were removed. A further 5830 records were excluded during title and abstract screening leaving 67 records for full-text review (see Figure 1). After full-text screening, 41 records were excluded due to the following reasons: 17 did not describe SNAPO risk outcomes; 9 were not RCTs; 6 utilised multicomponent interventions and/or comparators that did not allow for the effect of VR to be isolated; 6 studies did not meet the inclusion criteria of VR; 3 studies were duplicates or conference abstracts. The 26 records that remained were included. 

### 3.1. Study Characteristics

Of the 26 studies included, 5 assessed smoking cessation or reduction in smoking cigarettes [26,27,28,29,30], 12 solely focused on physical activity [31,32,33,34,35,36,37,38,39,40,41,42], 6 examined obesity and weight [43,44,45,46,47,48], 1 study examined both physical activity and obesity/weight-based outcomes [49], 1 study looked at nutrition in conjunction with obesity/weight outcomes [50], and 1 study focussed on obesity/weight, nutrition, and physical activity outcomes [51]. No studies studied the effectiveness of VR on alcohol consumption.

#### 3.1.1. Smoking Cessation or Reduction

All five studies that examined smoking behaviours were conducted with non-clinical populations. Two studies were conducted in the United States [26,30], one study in Argentina [27], one in Cyprus [28], and one in Spain [29]. Three studies targeted middle-aged adults, with mean age ranging from 39 years to 48 years [26,27,29]. The other two studies had younger samples, with mean ages of 22.44 years [28], and 16 years, respectively [30]. Three studies contained a majority of females [28,29,30], while two studies both consisted of 48% females [26,27]. Study duration varied, and were 21 days of treatment [27], 6 weeks [29], 7 weeks treatment [30], and 10 weeks of treatment [26]. One study reported that participants completed 6 sessions with a minimum of three maximum 30 days between each session [28]. Sample sizes ranged from 84 [28] to 136 [30]. The retention rates were 66% (55/84) at post-treatment after 6 sessions [28], 53% (46/86) at the end of a 10-week treatment program [28], 57% (68/120) at 90 days follow-up [27], 83% (85/102) at 12 months follow-up [29], and 73% (99/136) at 12 months follow-up [30]. A more detailed description of the study characteristics can be found in Supplementary Table S2.

In terms of the recruitment strategies adopted, one study advertised via their Facebook page and on national television [27], one study posted flyers around three universities and used classroom announcements [28], while another study used classroom presentations, posters, flyers, a sign-up table, school announcements, a posting in the school newspaper, and school liaison referrals to recruit students from 14 high schools [30]. One study recruited participants using flyers and advertising posted throughout the local community [29], whereas another study used referrals from area professionals, television and newspaper advertisements [26].

Three studies used fully immersive VR [26,27,29], and 2 studies used non-immersive VR [28,30]. Of the studies using fully immersive VR, one fitted the participant’s smartphone into a headset to access mindfulness sessions that apply exposure therapy that place participants in different virtual settings with smoking cues [27]. Similarly, the second fully immersive VR study had a therapist guide the participants through a virtual environment using cognitive behavioural therapy (CBT) techniques to respond to cravings, identify risks and triggers when exposed to smoking cues, in conjunction with Nicotine Replacement Therapy (NRT) [26]. The third study combined CBT with VR cue exposure where participants were fitted with VR eyewear and navigated through various virtual environments that contained smoking cues (e.g., others smoking, smoking paraphernalia) [29]. In contrast, one non-immersive VR study used avatar led acceptance and commitment therapy (ACT) sessions where participants would communicate with a virtual avatar on a computer screen [28] while one study used an internet-based virtual world that students could log into via a computer as their own avatar within a virtual “sky mall” and interact with different smoking related environments and have real-time discussions with each other and a smoking cessation counsellor who was in the world with them [30]. Of the fully immersive VR studies, one compared VR to a written self-help manual [27], another compared fully immersive VR plus CBT with just CBT [29], and one looked at it in conjunction with NRT in comparison to NRT by itself [26]. The two studies that used non-immersive VR technology used a waitlist control [28] and a no intervention control [30] respectively.

The smoking studies measured number of cigarettes smoked per day in the past week [26,27,28,30], abstinence at 24 h posttreatment [27,29], 7-day point-prevalence smoking abstinence [26,28,29,30], continuous abstinence at 1 month [29], 3 months [30], 6 months [29], and 12 months [29,30]. Some studies verified abstinence by measuring expired carbon monoxide (CO) levels [26,29], whereas others used self-reported questions [27,28,30]. 

#### 3.1.2. Physical Activity

Twelve studies analysed the effectiveness of VR on increasing levels of PA [31,32,33,34,35,36,37,38,39,40,41,42]. These studies were conducted in the Netherlands [34,36], Taiwan [32,33], Turkey [35,40], Spain [37,41], USA [38], Egypt [31], Switzerland [42] and the UK [39] (Supplementary Table S2).

Eight studies were conducted with clinical populations [31,32,33,35,36,39,41,42] from general inpatient or outpatients [42], to dementia or Alzheimer’s patients [36], children from a burn unit requiring physical rehabilitation [31], patients with Fibromyalgia (FM) [35,41], patients with multiple sclerosis [39], people who recently underwent cardiovascular (CV) surgery [32], or coronary artery bypass surgery [33]. In these studies with clinical populations, participants were recruited via the clinic or hospital they were attending [32,35,36,42], referred by physicians [33], mailed or handed an invitation letter [39], and using newspaper advertisements and word of mouth [36] and three studies did not report recruitment methods [31,35,41]. There were four studies conducted with non-clinical populations [34,37,38,40]. The trials with non-clinical populations recruited participants via an internet panel of residents [34], in person presentations and flyers given to people who attend nutritional clinics or had dropped out of their gym [37], and a mailout sent to all students attending a vocational school [40] while one study did not report recruitment methods [38]. Across all the PA studies, sample sizes ranged from 20 [35] to 958 [34]. Seven studies had a majority of females [31,34,35,37,39,40,41], three of which included only females [35,37,41]. One study included men only [32]. Across the studies mean age ranged from 13 [31] to 80 years old [36]. Studies ran for as short as a single session [42] to 3 weeks [37], 4 weeks with follow-up at week 8 [34,38], 8 weeks [35,40], 12 weeks [31,32,33], 12 weeks with follow-up at 24-weeks [36], 24 weeks with follow-up at week 48 [41], and 12 months [39].

Types of VR interventions varied between studies with 3 using fully immersive VR [35,40,41], 4 semi-immersive VR [32,33,36,42], and 5 non-immersive exergames or avatar studies [31,34,37,38,39]. The fully immersive VR technology used in these studies were head mounted displays and sensors to record movements [40], an Xbox Kinect and harness system to enable participants to deflect balls, move and dodge obstacles [35] and a specialised VR exergame (VirtualEx-FM) guided by a kinesiologist [41]. Of the 4 semi-immersive VR studies, three used a treadmill paired with a display screen that synced treadmill speeds and/or inclines with the virtual scene being presented either solo [32,33] or in a group of virtual walkers [42]. The remaining semi-immersive study used a ‘GameBike’ with pedal speeds synced to displays and followed a virtual city route paired with cognitive tasks [36]. In the 5 studies using non-immersive VR technology [31,34,37,38,39], exergaming with sensors tracking participant movements were used in conjunction with standard usual care/physical therapy in two clinical studies [31,39]. One clinical trial delivered the intervention using the Xbox Kinect system, where participants played a series of games [31], while another exergame used the Nintendo Wii system and Wii balance board to deliver the Mii-VitaliSe program, involving physiotherapist supported use of a variety of Nintendo Wii exercise games in the hospital and at home [39]. The study by Ruiz et al. [38] had participants view a photorealistic avatar of themselves performing 12 exercise routines in a virtual 3D environment exercise room. Similarly, Navarro et al. [37] used avatars with a photo realistic representation of the participant’s face, the avatar was either a representation of the participant’s ‘ideal’ self or used their current body dimensions in the other condition. One study [34] used an avatar delivered web-based PA intervention, whereby participants interacted with the virtual avatar using motivational interviewing techniques.

Comparators of the fully immersive VR studies were a group performing the same exercises without VR and a control group that avoided performing additional exercise except daily routine tasks [40], an exercise group performing aerobic training and Pilates [35], and a no activity control group [41]. For the semi-immersive studies, comparators included identical treadmill/cycling exercise without the VR display component [32,33,36], and running in front of a fixed image of lavender flowers [42]. Furthermore, one study also included a control group that performed relaxation and flexibility exercises [36]. Comparators and controls for non-immersive studies included a text-based intervention with identical content or no intervention [34], standard physical therapy [31], a 6-month wait list control [39], running in front of a fixed image corresponding to the virtual environment [37], an avatar representing an unknown person and a control group that observed static graphics depicting physical activity routines [38].

Five studies measured physical activity via self-report questionnaires [34,35,37,39,41]. One study used both self-report and objective measures of maximal heart rate [36]. Six studies used objective measures only to measure physical activity, exercise duration and exercise intensity such as maximal heart rate [33], Vo2 Peak [31,33], metabolic equivalent (METS) [33,42], accelerometer [38], activity monitored energy consumption [40], and Co2 saturation measured using a pulse oximeter [40]. Exercise duration [42] was measured in one study, as well as treadmill grades and speeds [32].

#### 3.1.3. Physical Activity and Obesity/Weight

Adamo et al.’s study [49] was conducted in Canada with 30 obese children aged between 12–17 years. The retention rate was 87% (26/30) at the end of the 10-week program. Of the 26 participants who completed the program, 60% were male, with a mean age of 13.9–15.1 years across the two experimental groups. Participants were recruited from the Endocrinology clinic at the Children’s Hospital of Eastern Ontario.

The study used semi-immersive VR in the form of a ‘GameBike’ with pedal speeds synced to displays and participants played a variety of race-based games [49]. The comparator group rode a stationary bike while listening to music [49]. The bike tracked participants’ distance travelled (km) and duration of the exercise session (mins). Participant peak heart rate (HR) was also assessed to measure exercise intensity and BMI was also collected.

#### 3.1.4. Obesity/Weight

Six studies assessed the effectiveness of VR on obesity/weight related outcomes [43,44,45,46,47,48] Three studies were conducted in Italy [44,46,47], 1 in Canada [48], 1 in the USA [43], and 1 in Brazil [45].

Four studies focused on clinical populations [44,45,46,47], two of which recruited consecutive patients seeking treatment for obesity [46] or an eating disorder [44]. Two studies were conducted with non-clinical populations who were recruited using advertisements [48] or mass emails and flyers [43]. Two studies did not report their recruitment methods [45,47]. 

Study samples ranged from 14 [48] to 163 [46] participants. Three studies included females only [44,46,47] and one study males only [48]. Among the remaining studies one had 98% female participants [43], the other had 40% females [45]. Across the studies age ranged from a mean of 23 years [48] to 69 years [45]. Intervention durations also varied lasting between 4 weeks [43], 5 weeks [47], three ran for 6 weeks [44,46,48], and 8 weeks [45]. Three of these studies also had follow-up sessions, one at 3 months [47], and two at 1-year [44,46]. Study retention rates were 86% (62/72) [45], 69% (113/163) [46], 60% (36/60) [47], 49% (44/90) [44], and 39% (36/92) [43] respectively. One study did not report retention rate [48].

Fully immersive VR was used in three studies [44,46,47]. Two studies used NeuroVR software and headsets to deliver a CBT program guided by a therapist through 14 virtual situations (e.g., gym, supermarket) [44,46]. One other study used fully immersive VR to provide relaxation training, where participants were asked to navigate around virtual environments while listening to an audio-narrative that ran through relaxation techniques [47]. One study used semi-immersive VR in the form of a ‘game bike’ that was linked to a screen displaying a range of virtual race-based games that were played by participant’s pedalling speed and steering [48]. Two studies used non-immersive VR, an exergames that used motion capture to replicate participant’s exercises in an avatar on screen [45], and the other used avatars in the virtual world to educate and promote physical activity [43].

The comparators/controls of the fully immersive VR studies were non-VR CBT sessions as a comparator and a standard behavioural program as a control [44,46], an imaginative condition that required participants to imagine the scenario [47], and a control condition that involved standard treatment for weight reduction [44,46,47]. The comparators for the semi-immersive study involved pedalling on a stationary bike [48]. The non-immersive VR study comparators/controls were a 2D social networking site where participants could interact and respond to health and nutrition posts and a no intervention control [43], and a functional training group that engaged in 10 physical activities, and another group that performed aerobic training on a stationary bicycle [45].

Obesity/weight was measured using objective measures such as scales to measure weight [43,44,46] and stadiometer to measure height [44,46]. One study used self-reported weight [47]. Two studies described using “standard procedures” [48] or did not say [45] how obesity/weight was measured.

#### 3.1.5. Obesity/Weight and Nutrition

This study was conducted with a clinical population that had completed the training phase of Cardiac Rehabilitation [50]. The study consisted of men only (2 women had dropped out) from Portugal, with a mean age of 55 to 59 years between groups. Forty-six participants were recruited from the Cardiovascular Prevention and Rehabilitation Unit; however, the method of recruitment was not detailed. The study ran for 6 months, and 72% of participants were retained at the end of the study.

This study used non-immersive VR through a Microsoft Kinect system that captured participants’ movements using sensors that were then translated to their in-game avatar [50]. The virtual physical therapists guided participants to perform a series of strength and endurance exercises, in conjunction participants in the experimental group also received a pamphlet with information on eating habits, smoking and physical activity [50]. For controls/comparators this study used an information booklet containing the same content as the intervention group, and a control group that received education on cardiovascular risk factors and were encouraged to walk daily [50]. 

Self-report measures were used to record nutritional outcomes, and height and weight were measured to record BMI.

#### 3.1.6. Obesity/Weight, Nutrition and Physical Activity

One study examined the effectiveness of VR on obesity/weight, nutrition, and PA with a non-clinical population [51]. Participants were from the USA, predominantly female (85%) with a mean age of 31 years. The sample size was 20 and sampling, recruitment methods, and retention rates were not reported. 

The study lasted 9 months and was split into two parts: the first 3 months of weight loss, then 6 months of weight maintenance. Non-immersive VR was used for the intervention through a computer program called Second Life in which participants controlled an avatar that could interact with other participants’ avatars and a health educator assisted with nutritional and weight-related strategies using a virtual location such as a kitchen or grocery store for education [51]. This trial used health educator delivered face-to-face meetings as a comparator.

A pedometer was used to record steps and self-report used for minutes exercised. Nutritional outcomes were recorded using self-report, and obesity outcomes using a digital scale to record weight, and stadiometer to measure height.

### 3.2. Primary Outcomes

#### 3.2.1. Effectiveness of VR on Smoking Cessation or Reduction

Five studies examined the effectiveness of VR on smoking cessation [27,28,29,30] or reducing number of cigarettes smoked [26,27,28,30]. Goldenhersch et al. [27] reported participants in the VR group were significantly more likely than those in the self-help manual control to have not smoked in the last 24 h (23% and 5%, respectively). Pericot-Valverde et al. [29] found no significant difference between VR plus CBT condition compared to CBT for expired CO verified 24 h point-prevalence abstinence posttreatment, nor did they find a significant difference for 7-day point-prevalence abstinence or continued abstinence at 1-month, 6 months, or 12 months. Another study [28] found a significant difference in self-reported 7-day point prevalence abstinence at posttreatment between the avatar-led acceptance and commitment therapy (ACT) program (51.9%) and a wait-list control (14.3%). Woodruff et al. [30] found a significant main effect in favour of the VR avatar condition over the no intervention control group on 7 day point prevalence abstinence at post-intervention, however, this did not persist at 3 months or 12 months follow-up.

Four studies examined the effectiveness of VR on number of cigarettes smoked [26,27,28,30]. Goldenhersch et al. [27] found that the VR group consumed significantly less cigarettes than the self-help manual control group at both week 3 of the intervention and postintervention. One study [28] also found those in the VR group (M = 2.89, SD = 4.01) smoked significantly less cigarettes per day than the control group (M = 7.61, SD = 7.26) at postintervention. Bordnick et al. [26] reported mean number of cigarettes smoked was significantly lower in the virtual reality skills training (VRST) group compared to the nicotine replacement therapy only (NRTO) group at posttreatment, 1-month, 2 months, and 6 months but not at 3 months. Woodruff et al.’s study [30] found significant main effects from baseline to postintervention in favour of the effectiveness of the VR avatar condition compared to the no intervention control group on number of cigarettes smoked per day in the last 7 days. However, across all four measurement periods (baseline, post-intervention, 3-month follow-up, and 12-month follow-up) there was no significant main effects between conditions for number of cigarettes smoked per day in the last 7 days, or the number of days smoked in the past 7 days [30]. Furthermore, Woodruff et al. [30] reported no between-group differences for each outcome at the 12-month follow-up.

#### 3.2.2. Effectiveness of VR on Physical Activity

Friederichs et al. [34] reported that at 1 month follow-up both the VR avatar-based group (M = 4.6, SD = 1.6) and the text-based comparator group (M = 4.7, SD = 1.8), were physically active for significantly more days than the no intervention control group (M = 4.0, SD = 1.9) [34]. The VR group and text group were not significantly different from each other [34]. Another study [42] found average exercise duration (minutes) was higher in the VR group (M = 11:12, SD = 2.54) compared to the control group (M = 8:54, SD = 2:39). A study with patients that had received coronary artery bypass grafting (CABG) [32] reported those in the VR treadmill condition (M = 4.64, SD = 1.4) were able to reach a significantly higher mean speed (mph) on the treadmill than the non-VR treadmill comparator group (M = 3.90, SD = 0.81). Ulas and Semine’s study [40], reported higher step counts in the traditional exercise group (M = 3398.59, SD = 529.19) compared to the fully immersive VR group (M = 2545.77, SD = 678.32). 

One study found no significant differences between the VR group and the stationary bike comparator group on percent of maximal heart rate as a measure of training intensity [36]. Similarly, another study with participants that were undergoing CABG [33] using the same VR treadmill treatment and non-VR treadmill comparator reported no significant difference in maximal HR between groups. However, this study [33] reported significantly higher Peak Vo2 and Peak METS in the VR group (Vo2 M = 22.47, SD = 4.48; METS M = 6.42, SD = 1.28) compared to the non-VR group (Vo2 M = 16.84, SD = 4.64; METS M = 4.81, SD = 1.33). Basha et al. [31] also showed a non-immersive VR group had significantly better Peak Vo2 (M = 30.1, SD = 2.22) than the control group (M = 26.85, SD = 1.42)). Ulas and Semine’s study [40] reported that those in the fully immersive VR group (M = 64.44, SD = 3.71) produced significantly lower METs during exercise, than the traditional exercise (TE) group (M = 68.34, SD = 5.90). In a study with participants with FM either using a specially developed exergame (VirtualEx-FM) with kinesiologist guided exercises, or a control group continuing daily life activities, METs per week were not significantly different [41]. Another study [42] with general inpatients and outpatients found those in the VR exercise treadmill group (M = 149, SD = 32) had significantly higher percentage of age-predicted METs achieved than the control group (M = 135, SD = 29).

Active energy consumption (Kcal) during exercise in one study [40] was significantly higher in the TE group (M = 360.72, SD = 53.28) compared to the VR group (M = 267.48, SD = 71.28). Ruiz et al. [38] found no significant difference in mean daily energy expenditure during PA between self-avatar, avatar representing someone else, or a static image group. 

The study by Karssemeijer et al. [36] with a clinical population showed no significant differences in Physical Activity Scale for the Elderly (PASE) scores between the VR stationary bike exergaming group, the aerobic stationary bike group, or the active control group at 12-weeks or 24-weeks. Gulsen et al.’s [35] study with FM patients compared the effectiveness of exercising in fully immersive VR plus group exercise with just group exercise at increasing level of PA and reported that those in the VR group (median = 1797.25) increased their International Physical Activity Questionnaire (IPAQ) PA scores significantly more than the exercise group (median = 528.50). A study by Navarro et al. [37] found no significant difference between avatars (ideal body dimensions or current body dimension) condition and a non-avatar condition on IPAQ scores. Thomas et al. [39] found the difference in Godin Leisure-Time Exercise Questionnaire (GLTEQ) scores (higher scores indicate higher PA) between the exergame VR group using the Nintendo Wii (M = 22.46, SD = 16.39) and the wait-list control (M = 11.20, SD = 9.77) produced a large effect size of 0.7 at 6 months.

#### 3.2.3. Effectiveness of VR on Physical Activity and Obesity/Weight

Adamo et al.’s study [49] showed that those using the interactive VR ‘Gamebike’ (M = 13.7, SD = 12.8) spent significantly less time (minutes) exercising at a vigorous intensity (80–100% peak HR) than the stationary bike music comparator group (M = 24.9, SD = 20.0). Adamo et al. [49] also reported no significant group differences for average minutes spent at moderate intensity (60–79% peak HR) and duration pedalled (minutes). Travel distance was also measured, revealing participants in the comparator group (M = 12.5, SD = 2.8) [49] travelled significantly further (Km) than the VR group (M = 10.3, SD = 2.2).

Obesity outcomes were measured in BMI (kg/m^2^), with results showing no significant difference on BMI at post intervention between the VR condition compared to riding a stationary bike while listening to music [49].

#### 3.2.4. Effectiveness of VR on Obesity/Weight

Obesity outcomes were measured in BMI (kg/m^2^) in 3 studies [44,45,48], 2 of which showed no significant difference on BMI between VR conditions compared to aerobic bicycle training at postintervention [45], and the stationary cycling condition at postintervention [48]. Cesa et al. [44] reported median BMI at 1-year follow-up was significantly lower in the VR group (36.2, SD = 5), than the CBT group (39.1, SD = 3.6), and inpatient program group (41.5, SD = 6). There was no significant difference between groups in weight loss at end of treatment [44]. However, there was a higher percentage of participants that lost further weight from posttreatment to 1-year follow-up in favour of both the VR group and the CBT group when compared with IP [44].

Three studies measured obesity outcomes using kilograms [46,47], or pounds [43], of these one showed significant between group differences [43]. Behm-Morawitz et al. [43] found those in the VR SL condition (M = 1.75) had significantly greater weight loss (pounds) at the end of the 4-week treatment, compared to the control condition using a 2D social networking site (M = 0.91). Manzoni et al. [46] found no significant differences in weight at posttreatment. Another study by Manzoni et al. [47] found no significant difference in weight loss (kg) between a fully immersive VR relaxation training protocol and both an imaginative condition and standard treatment control condition.

#### 3.2.5. Effectiveness of VR on Obesity/Weight and Nutrition

In the study by Vieira et al. [50] that examined the effectiveness of a home-based VR cardiac rehabilitation program, there was no significant difference on BMI and on most nutritional outcomes between VR compared to a paper booklet guide and minimal education control group at 3 months and 6 months. The only significant difference found favoured the booklet guide comparator group for mean triglycerides (mg/dL) at 6 months (M = 156, SD = 65.2), compared to the VR group (M = 104.1, SD = 38.2) and the control group (M = 100.6, SD= 14.0).

#### 3.2.6. Effectiveness of VR on Obesity/Weight, Nutrition and Physical Activity

Sullivan et al. [51] reported at 3 months mean percent weight loss was significantly greater in the face-to-face group (M = 10.8%, SD = 3.5%) than the VR group (M = 7.6%, SD = 5.1%). With regard to the nutritional outcomes, at 9 months those in the virtual program SL only condition (M = 2.7, SD = 0.7) self-reported having consumed significantly more fruit daily than those receiving face-to-face plus weight maintenance in SL (M = 1.9, SD = 0.4). However, self-reported daily consumption of vegetables was not significantly different between groups at 9 months [51]. Sullivan et al. [51] also measured weekly mean minutes of PA at 9 months and found no significant difference between the SLO and face-to-face group [51]. Those in the SLO group (M = 82,124, SD = 15,433) had a significantly higher step count per week at 9 months, than the face-to-face group (M = 59,903, SD = 12,405).

### 3.3. Secondary Outcomes

#### 3.3.1. Secondary Outcomes for Smoking Cessation or Reduction

One smoking cessation study [29] found the VR group attended 78% of sessions compared to 73.1% of sessions in the comparator group. 

#### 3.3.2. Secondary Outcomes for Physical Activity

Three PA studies measured participant satisfaction and acceptability of the intervention they received [31,34,36]. Two studies showed no significant differences between conditions, one in scores for overall appreciation and entertainment [34], the other in participant rating of training sessions [36]. The third study showed a significant difference between groups with the VR Xbox group providing a higher enjoyment rating score (M = 31.7, SD = 2.1) than the control group (M = 19.05, SD = 2.6) [31]. Two PA studies measured adherence rates [31,36], one of which showed a trend for those in the exergame group to be more likely to adhere to the program than an aerobic exercise group [36], the other showed no significant difference with 96% and 93% attendance for the VR group and the control group, respectively [31]. The Thomas et al. study [39] which used a Nintendo Wii console and physiotherapist, measured costs for these at 300 pounds (per unit) and 384 pounds (per participant), respectively. 

#### 3.3.3. Secondary Outcomes for Physical Activity and Obesity/Weight

The study by Adamo et al. [49] showed there was a significant difference in favour of the comparator group compared to the VR group with participants attending a mean of 92.3% and 86.1% of sessions, respectively.

#### 3.3.4. Secondary Outcomes for Obesity/Weight

In the study by Warburton et al. [48], mean percent of participant attendance was significantly higher in the VR group (78%) over the stationary cycling group (48%).

#### 3.3.5. Secondary Outcomes for Obesity/Weight and Nutrition

One study found no difference between the VR and comparator groups on adherence to the program [50].

#### 3.3.6. Secondary Outcomes for Obesity/Weight, Nutrition and Physical Activity

Sullivan et al. [51] found no difference between the VR and comparator groups on adherence to the program.

### 3.4. Quality Assessment

The results of the quality assessment for each of the studies can be seen in Table 1. Regarding global ratings, 3 studies were rated as moderate (2 obesity/weight trials [44,45] and 1 physical activity trial [31]), and the remaining 23 studies were rated as weak [26,27,28,29,30,32,33,34,35,36,37,38,39,40,41,42,43,46,47,48,49,50,51].

All studies received a weak rating for selection bias due to participation being under 60% and/or recruitment methods restricting representativeness of the target population. All studies were rated strong for study design as they were all RCTs or randomised trials. Blinding of participants and/or outcome assessors was not performed and therefore rated as weak in 19 studies [26,27,28,29,30,32,33,34,36,38,39,40,42,43,46,47,49,50,51] while 7 studies were rated as moderate for only blinding either the participants or assessors [31,35,37,41,44,45,48]. There were 9 studies rated as weak for data collection because there was no evidence of the measures being valid or reliable [27,28,30,35,36,39,41,47,51], 3 as moderate for using either valid or reliable measures [26,34,50], and 14 rated strong for using measures that were both valid and reliable [29,31,32,33,37,38,40,42,43,44,45,46,48,49]. Seven studies were rated weak for withdrawals and dropouts due to retention rates being below 60% [26,27,28,34,40,43,46,48,51], 6 as moderate (60–79% retention) [30,33,41,44,47,50], and 11 rated as strong (between 80–100% retention) [29,31,32,35,36,37,38,39,42,45,49]. Only 6 studies were rated weak for having less than 60% of confounders controlled for [30] or did not report controlling for potential confounders [34,37,39,43,46].

## 4. Discussion

### 4.1. Principal Findings

This systematic review is the first to examine the effectiveness of virtual reality technology on SNAPO risk factors and indicated that VR is a viable alternative and potentially more effective tool that can be used to treat smoking, poor nutrition, physical inactivity, and/or obesity. Thirteen studies showed VR conditions were more beneficial than the comparators used [26,27,28,30,31,32,33,34,35,39,42,43,44], nine showed VR was as effective as the comparator group [29,36,37,38,41,45,46,47,48], one study showed traditional exercise was more effective than the VR used [40] and three multiple health risks studies showed mixed results [49,50,51]. Given that VR-based treatments can be used independently at home it may remove barriers relating to travel distance, time constraints, or unique contexts such as COVID-19 restrictions. 

Three smoking related studies showed that VR was a more effective treatment option than controls/comparators for smoking cessation at posttreatment [27,28,30], but there was no difference at 1 to 12 months postintervention when measured with CBT or no intervention [29,30]. VR was found to be more effective at reducing the number of cigarettes people consumed at post-intervention [26,27,28,30], however only 1 study showed continued reduced cigarette consumption until 6 months follow up [26]. These results aligned with those found in other systematic reviews [16,20] whereby VR was beneficial in some studies, overall showing promise, however more studies with rigorous methodology are required to strengthen the evidence-base. For example, it was reported that VR-CET added no benefit to CBT but showed potential [16,20], and AAT-based VR and gamified behavioural and skill training were effective and appeared promising [16]. The current systematic review expands on previous work by affirming the potential for VR technology to be used to treat tobacco use. 

In relation to physical activity, this systematic review found mixed results on the effectiveness of VR-based interventions on increasing PA, and importantly, the majority of studies were conducted with clinical populations [31,32,33,35,36,39,41,42]. Just over half the studies (7/12) showed those in the VR group had significantly higher levels of PA compared to the control/comparator group [31,32,33,34,35,39,42]. Compared to a comparator/control, participants in the VR group were significantly more likely to spend time exercising [34,42], exercise at a higher intensity [32], and increase the level at which they exercise physically [35,39], or biochemically through peak Vo2 [31,33], and peak METS [33,42]. However, 4 studies found no between-group differences across these measures [36,37,38,41], and 1 study found significantly higher PA levels in a traditional exercise group compared to the fully immersive VR group [40]. The current review’s findings are similar to a review examining the use of VR in cardiac rehabilitation (CR) programs which found VR-based interventions increased physical activity [13].

This systematic review found the effectiveness of VR interventions on obesity compared to other treatments was inconclusive. One other review [15] reported the use of avatars to positively impact weight loss was promising, however the evidence was limited. This somewhat differs from the current review. While two studies showed VR conditions to be more effective at reducing BMI or weight (kg) than a 2D networking site [43], an in-patient program or CBT [44], four other studies found no between-group differences of the VR intervention and comparator/control groups [45,46,47,48]. Two studies compared VR enhanced CBT with CBT and a SBP, the study with people with a binge eating disorder [44] showed VR to be more effective at reducing weight, whereas the study with patients with morbid obesity found no between-group differences [46]. These differing results could be due to the variation in clinical populations in each study. 

To our knowledge, this systematic review was the first to examine the effectiveness of VR on multiple health risk behaviours. The review showed that one [51] of the three studies assessing multiple SNAPO risks (three studies assessed obesity [49,50,51], two PA [49,51], two nutrition [50,51]) demonstrated VR to be effective for some nutrition and PA outcomes but not other measures of PA and nutrition. Given the limited number of studies that have investigated the effectiveness of VR interventions on multiple health risk behaviours, future research should focus on addressing the limited evidence-base.

### 4.2. Secondary Outcomes

With regard to the secondary outcomes, seven studies [29,31,36,48,49,50,51] compared adherence to the programs between conditions, with one study finding participants in the VR program were significantly more likely to attend compared to a stationary cycling group [48]. Only one [31] of three studies [31,34,36] showed that participants were more accepting and satisfied with a PA VR intervention compared to a standard physical therapy protocol. Furthermore, only one study assessed the costs of a PA VR program using Nintendo Wii compared to a physiotherapist, and reported 300 pounds per Wii unit and 384 pounds for a physiotherapist per participant [39]. Overall, it appears VR interventions were no more effective than controls or comparators for these secondary outcomes. However, more studies measuring these outcomes are needed to advance the literature.

### 4.3. Quality Assessment

In relation to methodological quality only three studies were rated as moderate on the global rating [31,44,45], while 23 studies received a weak rating [26,27,28,29,30,32,33,34,35,36,37,38,39,40,41,42,43,46,47,48,49,50,51]. This was primarily due to studies rating poorly in selection bias and blinding. Future research should aim to recruit using random sampling from broader populations, and while blinding of participants can be difficult, researchers should aim to blind outcome assessments when possible. Future research should also aim to improve retention rates, and strategies such as providing incentives for study completion appear promising [52].

### 4.4. Strengths and Limitations of Review

This review had several strengths including a comprehensive search of five databases and the inclusion of randomised trials only which provides confidence in these findings. Furthermore, screening, data extraction and quality assessment was conducted independently by two researchers with a third researcher consulted if discrepancies could not be resolved which supports the rigorous approach used during this review.

Limitations of this review included that the quality assessments were based on the information in the published paper and authors were not contacted to provide additional information that may have been omitted from the paper. Furthermore, non-English studies were excluded potentially limiting the generalisability of the review findings. While not a limitation of this review’s methodology, the absence of studies on alcohol consumption and the disproportionate number of studies across smoking, nutrition, PA, and obesity limits the conclusions that can be made.

### 4.5. Implications for Practice across SNAPO Risks

This review has outlined the potential viability of VR interventions as an alternative method of treatment for tobacco smoking, nutrition, PA, and obesity. VR-based research is more established for some behaviours compared to others, with most studies relating to PA. VR is becoming more accessible and portable and so could allow for greater use in the home or clinic as a potential alternative for modifying health risk behaviours. Examination of the demographics across the studies emphasises the potential use of VR interventions across different age groups between 10–80 years, countries, and gender. 

### 4.6. Future VR Intervention Research across SNAPO Risks

With continuous improvements to VR technology and increased accessibility with gaming consoles in the home, there is an opportunity for future research particularly relating to alcohol consumption and nutrition, as well as smoking and obesity.

There is also a lack of studies with younger populations with very few studies in this review including participants under 25 years [28,30,31,40,43,48,49]. More randomised trials with longer follow-up assessments are also needed to understand the long-term effectiveness of VR interventions on SNAPO risk factors. Furthermore, additional studies need to be conducted with non-clinical populations, and a more balanced representation of males to females is needed particularly for studies relating to obesity.

All types (fully, semi-, and non-immersive) of VR interventions appear to be viable options for treating smoking, PA, obesity, or nutrition. Given VR technology is constantly improving, and fully immersive VR is relatively new, future research should continue to study the effectiveness of this technology on improving SNAPO risk factors. Furthermore, as more studies utilise each level of immersive VR, future research could assess whether effectiveness varies by the type of VR technology used.

Future research investigating the effectiveness of VR interventions on SNAPO risks should also examine other important features such as costs, satisfaction, or usage to identify the feasibility and practicality of implementing a VR program.

## 5. Conclusions

Overall, this review demonstrated that VR interventions have the potential to be an effective alternative to current smoking, nutrition, PA and obesity treatments and that VR is a promising intervention within healthcare settings. Further research is required to expand the evidence-base examining the effectiveness of VR interventions across SNAPO risks, particularly for alcohol consumption, nutrition, smoking and obesity.

## Figures and Tables

**Table 1:** Quality assessment of included studies.

	Selection Bias	Study Design	Confounders	Blinding	Data Collection	Withdrawals and Dropouts	Global Rating
**Smoking**							
Bordnick et al. [26]	Weak	Strong	Strong	Weak	Moderate	Weak	Weak
Goldenhersch et al. [27]	Weak	Strong	Strong	Weak	Weak	Weak	Weak
Karekla et al. [28]	Weak	Strong	Strong	Weak	Weak	Weak	Weak
Pericot-Valverde et al. [29]	Weak	Strong	Strong	Weak	Strong	Strong	Weak
Woodruff et al. [30]	Weak	Strong	Weak	Weak	Weak	Moderate	Weak
**Physical Activity**							
Basha et al. [31]	Weak	Strong	Strong	Moderate	Strong	Strong	Moderate
Chuang et al. [32]	Weak	Strong	Strong	Weak	Strong	Strong	Weak
Chuang et al. [33]	Weak	Strong	Strong	Weak	Strong	Moderate	Weak
Friederichs et al. [34]	Weak	Strong	Weak	Weak	Moderate	Weak	Weak
Gulsen et al. [35]	Weak	Strong	Strong	Moderate	Weak	Strong	Weak
Karssemeijer et al. [36]	Weak	Strong	Strong	Weak	Weak	Strong	Weak
Navarro et al. [37]	Weak	Strong	Weak	Moderate	Strong	Strong	Weak
Ruiz et al. [38]	Weak	Strong	Strong	Weak	Strong	Strong	Weak
Thomas et al. [39]	Weak	Strong	Weak	Weak	Weak	Strong	Weak
Ulas and Semin [40]	Weak	Strong	Strong	Weak	Strong	Weak	Weak
Villafaina et al. [41]	Weak	Strong	Strong	Moderate	Weak	Moderate	Weak
Wilzeck et al. [42]	Weak	Strong	Strong	Weak	Strong	Strong	Weak
**Physical Activity and Obesity/Weight**							
Adamo et al. [49]	Weak	Strong	Strong	Weak	Strong	Strong	Weak
**Obesity/Weight**							
Behm-Morawitz et al. [43]	Weak	Strong	Weak	Weak	Strong	Weak	Weak
Cesa et al. [44]	Weak	Strong	Strong	Moderate	Strong	Moderate	Moderate
Ferraz et al. [45]	Weak	Strong	Strong	Moderate	Strong	Strong	Moderate
Manzoni et al. [46]	Weak	Strong	Weak	Weak	Strong	Weak	Weak
Manzoni et al. [47]	Weak	Strong	Strong	Weak	Weak	Moderate	Weak
Warburton et al. [48]	Weak	Strong	Strong	Moderate	Strong	Weak	Weak
**Obesity/Weight and Nutrition**							
Vieira et al. [50]	Weak	Strong	Strong	Weak	Moderate	Moderate	Weak
**Obesity/Weight, Nutrition and Physical Activity**							
Sullivan et al. [51]	Weak	Strong	Strong	Weak	Weak	Weak	Weak

## Data Availability

Not applicable.

## Supplementary Materials

The following supporting information can be downloaded at: https://www.mdpi.com/article/10.3390/ijerph191710821/s1. Table S1: Search strategy for MEDLINE, Embase, PsycInfo, CENTRAL, and Scopus; Table S2: Characteristics of studies examining the effectiveness of VR interventions on smoking, nutrition, physical activity and/or obesity/weight risk factors.

## References

[1] World Health Organization (2021). Noncommunicable Diseases. https://www.who.int/news-room/fact-sheets/detail/noncommunicable-diseases.

[2] Australian Institute of Health and Welfare (2021). Australian Burden of Disease Study 2018—Key Findings.

[3] World Health Organization (2022). Tobacco. https://www.who.int/news-room/fact-sheets/detail/tobacco.

[4] Afshin A., Sur P.J., Fay K.A., Cornaby L., Ferrara G., Salama J.S., Mullany E.C., Abate K.H., Abbafati C., Abebe Z. (2019). Health effects of dietary risks in 195 countries, 1990–2017: A systematic analysis for the Global Burden of Disease Study 2017. Lancet.

[5] World Health Organization (2022). Alcohol. https://www.who.int/news-room/fact-sheets/detail/alcohol.

[6] Ritchie H., Roser M. (2017). Obesity. Our World in Data. https://ourworldindata.org/obesity.

[7] Li L., Yu F., Shi D., Shi J., Tian Z., Yang J., Wang X., Jiang Q. (2017). Application of virtual reality technology in clinical medicine. Am. J. Transl. Res..

[8] Heizenrader L.L.C. (2019). The 3 Types of Virtual Reality. https://heizenrader.com/the-3-types-of-virtual-reality/#:~:text=There%20are%203%20primary%20categories,%2C%20and%20fully%2Dimmersive%20simulations.

[9] Corregidor-Sánchez A.I., Sergura-Fragoso A., Rodriguez-Hernández M., Criado-Alvarez J.J., González-Gonzalez J., Polonio-López B. (2020). Can exergames contribute to improving walking capacity in older adults? A systematic review and meta-analysis. Maturitas.

[10] Corregidor-Sánchez A.I., Sergura-Fragoso A., Rodriguez-Hernández M., Jiménez-Rojas C., Polonio-López B., Criado-Álvarez J.J. (2021). Effectiveness of virtual reality technology on functional mobility of older adults: Systematic review and meta-analysis. Age Ageing.

[11] Durl J., Dietrich T., Pang B., Potter L., Carter L. (2018). Utilising virtual reality in alcohol studies: A systematic review. Health Educ. J..

[12] Felipe F.A., de Carvalho F.O., Silva E.R., Santos N.G.L., Fontes P.A., de Almeida A.S., Garção D.C., Nunes P.S., Araújo A.A.S. (2020). Evaluation instruments for physical therapy using virtual reality in stroke patients: A systematic review. Physiotherapy.

[13] García-Bravo S., Cuesta-Gómez A., Campuzano-Ruiz R., López-Navas M.J., Domínguez-Paniagua J., Araújo-Narváez A., Barreñada-Copete E., García-Bravo C., Flórez-García M.T., Botas-Rodríguez J. (2021). Virtual reality and video games in cardiac rehabilitation programs. A systematic review. Disabil. Rehabil..

[14] Ghiţă A., Gutiérrez-Maldonado J. (2018). Applications of virtual reality in individuals with alcohol misuse: A systematic review. Addict. Behav..

[15] Horne M., Hill A., Murells T., Ugail H., Irving, Chinnadorai R., Hardy M. (2020). Using avatars in weight management settings: A systematic review. Internet Interv..

[16] Keijsers M., Vega-Corredor M.C., Tomintz M., Hoermann S. (2021). Virtual reality technology use in cigarette craving and smoking interventions (i “virtually” quit): Systematic review. J. Med. Internet Res..

[17] Laver K.E., George S., Thomas S., Deutsch J.E., Crotty M. (2017). Virtual reality for stroke rehabilitation. Cochrane Database Syst. Rev..

[18] Miller K.J., Adair B.S., Pearce A.J., Said C.M., Ozanne E., Morris M.M. (2014). Effectiveness and feasibility of virtual reality and gaming system use at home by older adults for enabling physical activity to improve health-related domains: A systematic review. Age Ageing.

[19] Peng W., Crouse J.C., Lin J.H. (2013). Using active video games for physical activity promotion: A systematic review of the current state of research. Health Educ. Behav..

[20] Segawa T., Baudry T., Bourla A., Blanc J., Peretti C., Mouchabac S., Ferreri F. (2020). Virtual reality (VR) in assessment and treatment of addictive disorders: A systematic review. Front. Neurosci..

[21] Trahan M.H., Maynard B.R., Smith K.S., Farina A.S.J., Khoo Y.M. (2019). Virtual reality exposure therapy on alcohol and nicotine: A systematic review. Res. Soc. Work. Pract..

[22] Tsamitros N., Sebold M., Gutwinski S., Back A. (2021). Virtual reality-based treatment approaches in the field of substance use disorders. Curr. Addict. Rep..

[23] Page M.J., McKenzie J.E., Bossuyt P.M., Boutron I., Hoffman T.C., Mulrow C.D., Shamseer L., Tetzlaff J.M., Akl E.A., Brennan S.E. (2021). The PRISMA 2020 statement: An updated guideline for reporting systematic reviews. BMJ.

[24] Effective Public Health Practice Project (2010). Quality Assessment Tool for Quantitative Studies. https://www.ephpp.ca/PDF/Quality%20Assessment%20Tool_2010_2.pdf.

[25] Effective Public Health Practice Project (2009). Quality Assessment Tool for Quantitative Studies Dictionary. https://www.ephpp.ca/PDF/QADictionary_dec2009.pdf.

[26] Bordnick P.S., Traylor A.C., Carter B.L., Graap K.M. (2012). A feasibility study of virtual reality-based coping skills training for nicotine dependence. Res. Soc. Work. Pract..

[27] Goldenhersch E., Thrul J., Ungaretti J., Rosencovich N., Waitman C., Ceberio M.R. (2020). Virtual reality smartphone-based intervention for smoking cessation: Pilot randomized controlled trial on initial clinical efficacy and adherence. J. Med. Internet Res..

[28] Karekla M., Savvides S.N., Gloster A. (2021). An avatar-led intervention promotes smoking cessation in young adults: A pilot randomized clinical trial. Ann. Behav. Med..

[29] Pericot-Valverde I., Secades-Villa R., Gutierrez-Maldonado J. (2019). A randomized clinical trial of cue exposure treatment through virtual reality for smoking cessation. J. Subst. Abus. Treat..

[30] Woodruff S.I., Conway T.L., Edwards C.C., Elliott S.P., Crittenden J. (2007). Evaluation of an internet virtual world chat room for adolescent smoking cessation. Addict. Behav..

[31] Basha M.A., Aboelnour N.H., Aly S.M., Kamel F.A.H. (2020). Impact of kinect-based virtual reality training on physical fitness and quality of life in severely burned children: A monocentric randomized controlled trial. Ann. Phys. Rehabil. Med..

[32] Chuang T.Y., Sung W.H., Chang H.A., Wang R.Y. (2006). Effect of a virtual reality-enhanced exercise protocol after coronary artery bypass grafting. Phys. Ther..

[33] Chuang T.Y., Sung W.H., Lin C.Y. (2005). Application of a virtual reality-enhanced exercise protocol in patients after coronary bypass. Arch. Phys. Med. Rehabil..

[34] Friederichs S., Bolman C., Oenema A., Guyaux J., Lechner L. (2014). Motivational interviewing in a Web-based physical activity intervention with an avatar: Randomized controlled trial. J. Med. Internet Res..

[35] Gulsen C., Soke F., Eldemire K., Apaydin Y., Ozkul C., Guclu-Gunduz A., Akcali D.T. (2022). Effect of fully immersive virtual reality treatment combined with exercise in fibromyalgia patients: A randomized controlled trial. Assist. Technol..

[36] Karssemeijer E.G.A., Bosser W.J., Aaronson J.A., Sanders L.M.J., Kessels R.P.C., Rikkert M.G.M.O. (2019). Exergaming as a physical exercise strategy reduces frailty in people with dementia: A randomized controlled trial. J. Am. Med. Dir. Assoc..

[37] Navarro J., Cebolla A., Llorens R., Borrego A., Baños R.M. (2020). Manipulating self-avatar body dimensions in virtual worlds to complement an internet-delivered intervention to increase physical activity in overweight women. Int. J. Environ. Res. Public Health.

[38] Ruiz J.G., Andrade A.D., Anam R., Aguiar R., Sun H., Roos B.A. (2012). Using anthropomorphic avatars resembling sedentary older individuals as models to enhance self-efficacy and adherence to physical activity: Psychophysiological correlates. Stud. Health Technol. Inform..

[39] Thomas S., Fazakarley L., Thomas P.W., Collyer S., Brenton S., Perring S., Scott R., Thomas F., Thomas C., Jones K. (2017). Mii-vitaliSe: A pilot randomised controlled trial of a home gaming system (Nintendo Wii) to increase activity levels, vitality and well-being in people with multiple sclerosis. BMJ Open.

[40] Ulas K., Semin I. (2021). The biological and motivational effects of aerobic exercise with virtual reality. Res. Q. Exerc. Sport.

[41] Villafaina S., Borrega-Mouquinho Y., Fuentes-García J.P., Collado-Mateo D., Gusi N. (2020). Effect of exergame training and detraining on lower-body strength, agility, and cardiorespiratory fitness in women with fibromyalgia: Single-blinded randomized controlled trial. Int. J. Environ. Res. Public Health.

[42] Wilzeck V.C., Hufschmid J., Bischof L., Hansi C., Nägele M.P., Beer J.J., Hufschmid U. (2020). A significant increase in exercise test performance with virtual group motivation: A randomised open-label controlled trial. Swiss Med. Wkly..

[43] Behm-Morawitz E., Lewallen J., Choi G. (2016). A second chance at health: How a 3D virtual world can improve health self-efficacy for weight loss management among adults. Cyberpsychology Behav. Soc. Netw..

[44] Cesa G.L., Manzoni G.M., Bacchetta M., Castelnuovo G., Conti S., Gaggioli A., Mantovani F., Molinari E., Cárdenas-López G., Riva G. (2013). Virtual reality for enhancing the cognitive behavioral treatment of obesity with binge eating disorder: Randomized controlled study with one-year follow-up. J. Med. Internet Res..

[45] Ferraz D.D., Trippo K.V., Duarte G.P., Neto M.G., Santos K.O.B., Filho J.O. (2018). The effects of functional training, bicycle exercise, and exergaming on walking capacity of elderly patients with Parkinson disease: A pilot randomized controlled single-blinded trial. Arch. Phys. Med. Rehabil..

[46] Manzoni G.M., Cesa G.L., Bacchetta M., Castelnuovo G., Conti S., Gaggioli A., Mantovani F., Molinari E., Cárdenas-López G., Riva G. (2016). Virtual reality-enhanced cognitive-behavioral therapy for morbid obesity: A randomized controlled study with 1 year follow-up. Cyberpsychology Behav. Soc. Netw..

[47] Manzoni G.M., Pagnini F., Gorini A., Preziosa A., Castelnuovo G., Molinari E., Riva G. (2009). Can relaxation training reduce emotional eating in women with obesity? An exploratory study with 3 months of follow-up. J. Am. Diet. Assoc..

[48] Warburton D.E.R., Bredin S.S.D., Horita L.T.L., Zbogar D., Scott J.M., Esch B.T.A., Rhodes R.E. (2007). The health benefits of interactive video game exercise. Appl. Physiol. Nutr. Metab..

[49] Adamo K.B., Rutherford J.A., Goldfield G.S. (2010). Effects of interactive video game cycling on overweight and obese adolescent health. Appl. Physiol. Nutr. Metab..

[50] Vieira A.S.D.S., de Melo M.C.D.A., Noites S.P.A.R.S., Machado J., Gabriel M.M. (2017). The effect of virtual reality on a home-based cardiac rehabilitation program on body composition, lipid profile and eating patterns: A randomized controlled trial. Eur. J. Integr. Med..

[51] Sullivan D.K., Goetz J.R., Gibson C.A., Washburn R.A., Smith B.K., Lee J., Gerald S., Fincham T., Donnelly J.E. (2013). Improving weight maintenance using virtual reality (second life). J. Nutr. Educ. Behav..

[52] Brueton V.C., Tierney J.F., Stenning S., Meredith S., Harding S., Nazareth I., Rait G. (2014). Strategies to improve retention in randomised trials: A Cochrane systematic review and meta-analysis. BMJ Open.

