# Supplementary material for: The Effectiveness of Virtual Reality Interventions on Smoking, Nutrition, Alcohol, Physical Activity and/or Obesity Risk Factors: A Systematic Review
Source: Int J Environ Res Public Health. 2022 Aug 30;19(17):10821. doi: 10.3390/ijerph191710821 (PMC9517886; doi:10.3390/ijerph191710821)
Supplement: Supplementary file 1 [file ijerph-19-10821-s001.zip › Supplementary Table S1.pdf]

Supplementary Table S1: Search strategy for MEDLINE, Embase, PsycInfo, CENTRAL, and Scopus

MEDLINE and Embase	
1	exp virtual reality/
2	exp augmented reality/
3	exp computer simulation/
4	VR.mp
5	Virtual realit*.mp.
6	simulated 3-D environment.mp
7	monoscopic video.mp
8	stereoscopic video.mp
9	avatar*.mp
10	1 or 2 or 3 or 4 or 5 or 6 or 7 or 8 or 9
11	exp Smoking/
12	exp Smoking Cessation/
13	exp Tobacco/
14	exp Tobacco Products/
15	exp “Tobacco Use”/
16	exp "Tobacco Use Cessation"/
17	exp Nicotine/
18	smok*.mp
19	tobacco.mp
20	cigar*.mp
21	exp Alcohol Drinking/
22	exp Drinking Behavior/
23	exp Alcoholism/
24	alcohol*.mp
25	alcohol abuse.mp.
26	exp Diet/
27	exp Diet, Healthy/

28	exp Diet, Food, and Nutrition/
29	exp Fruit/
30	exp Vegetables/
31	diet*.mp
32	nutrition.mp
33	exp Exercise/
34	exp Sedentary Behavior/
35	"physical activit*".mp
36	"physical inactiv*".mp
37	exp Obesity/
38	exp Overweight/
39	exp Body Weight/
40	exp Weight Loss/
41	obes*.mp
42	weight.mp
43	"body mass".mp
44	11 or 12 or 13 or 14 or 15 or 16 or 17 or 18 or 19 or 20 or 21 or 22 or 23 or 24 or 25 or 26 or 27 or 28 or 29 or 30 or 31 or 32 or 33 or 34 or 35 or 36 or 37 or 38 or 39 or 40 or 41 or 42 or 43
45	exp Randomized Controlled Trial/
46	exp Randomized Controlled Trials as Topic/
47	exp Clinical Trial/
48	exp Clinical Trials as Topic/
49	exp Pragmatic Clinical Trial/
50	exp Pragmatic Clinical Trials as Topic/
51	exp Random Allocation/
52	random*.mp
53	RCT*.mp
54	45 or 46 or 47 or 48 or 49 or 50 or 51 or 52 or 53
55	10 and 44 and 54

PsycInfo	
1	exp virtual reality/
2	exp augmented reality/
3	exp computer simulation/
4	VR.mp
5	Virtual realit*.mp.
6	simulated 3-D environment.mp
7	stereoscopic video.mp
8	monoscopic video.mp
9	avatar*.mp
10	1 or 2 or 3 or 4 or 5 or 6 or 7 or 8 or 9
11	exp Smoking Cessation/
12	exp "Tobacco Use"/
13	exp Nicotine/
14	smok*.mp
15	tobacco.mp
16	cigar*.mp
17	exp Drinking Behavior/
18	exp Alcoholism/
19	alcohol*.mp
20	alcohol abuse.mp.
21	exp Fruit/
22	diet*.mp
23	nutrition.mp
24	exp Exercise/
25	exp Sedentary Behavior/
26	"physical activit*".mp
27	"physical inactiv*".mp
28	exp Obesity/
29	exp Overweight/
30	exp Body Weight/

31	exp Weight Loss/
32	obes*.mp
33	weight.mp
34	“body mass”.mp
35	11 or 12 or 13 or 14 or 15 or 16 or 17 or 18 or 19 or 20 or 21 or 22 or 23 or 24 or 25 or 26 or 27 or 28 or 29 or 30 or 31 or 32 or 33 or 34
36	exp Randomized Controlled Trial/
37	exp Clinical Trial/
38	random*.mp
39	RCT*.mp
40	36 or 37 or 38 or 39
41	10 and 35 and 40
**CENTRAL**	
1	MeSH descriptor: [Virtual Reality] explode all trees
2	(virtual NEXT (realit* or environment* or world* or self or avatar)):ti,ab,kw OR (avatar*):ti,ab,kw OR (VR):ti,ab,kw OR ((monoscopic or stereoscopic) NEXT video):ti,ab,kw OR ("simulated 3D environment"):ti,ab,kw (Word variations have been searched)
3	MeSH descriptor: [Augmented Reality] explode all trees
4	MeSH descriptor: [Computer Simulation] explode all trees
5	1 or 2 or 3 or 4
6	MeSH descriptor: [Smoke] explode all trees
7	MeSH descriptor: [Smoking Cessation] explode all trees
8	MeSH descriptor: [Tobacco] explode all trees
9	MeSH descriptor: [Tobacco Products] explode all trees
10	MeSH descriptor: [Tobacco Use] explode all trees
11	MeSH descriptor: [Tobacco Use Cessation] explode all trees
12	MeSH descriptor: [Nicotine] explode all trees
13	(smok*):ti,ab,kw OR (tobacco):ti,ab,kw OR (cigar*):ti,ab,kw
14	MeSH descriptor: [Alcohols] explode all trees
15	MeSH descriptor: [Drinking Behavior] explode all trees

16	MeSH descriptor: [Alcohols] explode all trees
17	(alcohol*):ti,ab,kw OR (alcohol NEXT (abuse or misuse or addiction*)):ti,ab,kw
18	MeSH descriptor: [Diet] explode all trees
19	MeSH descriptor: [Diet, Healthy] explode all trees
20	MeSH descriptor: [Diet, Food, and Nutrition] explode all trees
21	MeSH descriptor: [Fruit] explode all trees
22	MeSH descriptor: [Vegetables] explode all trees
23	(diet*):ti,ab,kw OR (nutrition):ti,ab,kw
24	MeSH descriptor: [Exercise] explode all trees
25	MeSH descriptor: [Sedentary Behavior] explode all trees
26	(physical NEXT (activit* or inactiv* or exercise)):ti,ab,kw
27	MeSH descriptor: [Obesity] explode all trees
28	MeSH descriptor: [Overweight] explode all trees
29	MeSH descriptor: [Weight Loss] explode all trees
30	(obes*):ti,ab,kw OR (weight):ti,ab,kw OR ("body mass"):ti,ab,kw
31	6 or 7 or 8 or 9 or 10 or 11 or 12 or 13 or 14 or 15 or 16 or 17 or 18 or 19 or 20 or 21 or 22 or 23 or 24 or 25 or 26 or 27 or 28 or 29 or 30
32	MeSH descriptor: [Randomized Controlled Trial] explode all trees
33	MeSH descriptor: [Randomized Controlled Trials as Topic] explode all trees
34	MeSH descriptor: [Clinical Trial] explode all trees
35	MeSH descriptor: [Clinical Trials as Topic] explode all trees
36	MeSH descriptor: [Pragmatic Clinical Trial] explode all trees
37	MeSH descriptor: [Pragmatic Clinical Trials as Topic] explode all trees
38	MeSH descriptor: [Random Allocation] explode all trees
39	(random*):ti,ab,kw OR (RCT*):ti,ab,kw
40	32 or 33 or 34 or 35 or 36 or 37 or 38 or 39
41	5 and 31 and 40
**Scopus**	
VR or virtual* or "virtual reality" or "augmented reality" or avatar AND	
RCT or random* or "randomised control*" or "control trial" or "clinical control*" or “cluster randomised trial” AND	

Smok* or Smoking or cigarette or "smoking cessation" or tobacco or nicotine OR
Alcohol or "Alcohol use disorder" or "substance abuse" or alcohol* or "substance abuse" OR
nutrition* or "healthy eating" or diet* or "healthy food" or nutrient or "fruit and vegetable" OR
"weight loss" or exercise or "physical activity" or weigh* or obesity or overweight or "body weight"
