# Supplementary material for: The Effectiveness of Virtual Reality Interventions on Smoking, Nutrition, Alcohol, Physical Activity and/or Obesity Risk Factors: A Systematic Review
Source: Int J Environ Res Public Health. 2022 Aug 30;19(17):10821. doi: 10.3390/ijerph191710821 (PMC9517886; doi:10.3390/ijerph191710821)
Supplement: Supplementary file 1 [file ijerph-19-10821-s001.zip › Supplementary Table S2.pdf]

Supplementary Table S2: Characteristics of studies examining the effectiveness of VR interventions on smoking, nutrition, physical activity and/or obesity/weight risk factors

Authors<br>*Country*<br>*Years data collected*	*Setting*<br>*Sample characteristics (sample size, demographics)*	*Recruitment method*<br>*Eligibility criteria*	*Study design*<br>*Virtual reality intervention description*<br>*Control/comparators description*<br>*Retention at follow-up/end of treatment*	*Measures of health behaviours*	*Health behaviours outcomes*	*Secondary Outcomes*<br><br>*Use of support received*<br>*Satisfaction/acceptability of support received*<br>*Costs*
**Smoking**						
Bordnick et al. [26]<br>USA<br>NA	Nicotine dependent treatment seeking cigarette smokers in Houston USA, (n=86)<br><br>Baseline demographics obtained (n=46)<br>**Age (mean, SD)**<br>NRTO=46.2, 8.4<br>VRST=47.9,10.4<br>**Sex**<br>48% (22/46) female<br>**Ethnicity**<br>85% (39/46) African American<br>11% (5/46) Caucasian<br>4% (2/46) Hispanic	Nicotine-dependent treatment-seeking cigarette smokers, referred by area professionals or recruited through television and newspaper advertisements.<br><br>**Eligibility criteria**<br>Diagnostic and Statistical Manual of Mental Disorders—Fourth Edition (DSM-IV) diagnosis of nicotine dependence, being in self-reported good physical health and between 18 and 70 years, having a sixth grade or higher English reading level.<br><br>Exclusion criteria included DSM-IV diagnosis of severe mental illness or other substance dependence (nicotine not included), current use of	**Study design**<br>Two-group randomised design<br><br>10-week treatment groups:<br>**Intervention**<br>N=44<br>*Virtual reality skills training (VRST) + nicotine replacement therapy (NRT):*<br>Attended 1 hr sessions weekly for 10 weeks. VRST sessions were manual based, individualized (e.g., preferred brand of cigarettes used in VR sessions), and conducted by a graduate-level trained therapist. The VRST intervention combined CBT techniques and VR-augmented cue exposure skills training conducted in VR. During a typical VR session, while the participant was immersed in the environment, the therapist used the VR scenario to assess and teach coping skills. For example, the therapist would use the virtual party (with smokers) to help the participant identify high-risk triggers and social situations that elicit a strong desire to smoke. After triggers were identified, the therapist used the virtual party to teach skills and strategies (e.g., managing	**Smoking**<br>*Expired CO levels:* Smoking abstinence (smoked in the previous 7 days)<br><br>*Number of cigarettes smoked at each study phase*: Timeline follow back was used to assess the number of cigarettes smoked per day.	**Smoking Outcomes**<br>VRST treatment group reported smoking significantly fewer cigarettes in the previous 7 days compared to the NRTO control group (at 1, 2, and 6-month follow-up), F(1, 44) = 4.4, p < .05, which was confirmed with expired CO levels taken at the same time point, NRTO (M = 2.4, SD = .3.2) and VRST (M = .65, SD = 1.2), F(1, 44) = 4.24, p < .05, ηp 2 = .14.<br><br>*Cigarettes smoked (m, SD)*<br>End of treatment<br>NRTO (n=25) = 2.4, 3.2<br>VRST (n=21) = 0.65, 1.2<br>S (p<.05)<br><br>1 month<br>NRTO (n=25) = 3.3, 4.0	NA

Authors Country Years data collected	*Setting Sample characteristics (sample size, demographics)*	*Recruitment method Eligibility criteria*	*Study design Virtual reality intervention description Control/comparators description Retention at follow-up/end of treatment*	*Measures of health behaviours*	*Health behaviours outcomes*	*Secondary Outcomes<br><br>Use of support received Satisfaction/acceptability of support received Costs*
		psychotropic medications, pregnancy, history of seizures or seizure disorders, use of illicit drugs in the past 30 days, or other significant health problems that would preclude participation.	craving, planning an exit strategy) to cope effectively with the situation.<br>The VRST therapist guided participants in the VR scenarios and controlled all interactions and stimuli through a real-time computer interface.<br><br>All VR scenarios used advanced three-dimensional (3D) computer graphics; 3D models with photo-realistic textures; integration of people for social interactions; spatial surround sound audio cues; and scents, including cigarette smoke, beer, pizza, and coffee, to produce interactive, immersive smoking scenarios that resemble real-world environments and social interactions.<br><br>**Control/Comparator**<br>N=42<br>*Nicotine Replacement Therapy only (NTRO):* Attended 10 weekly clinic visits to pick up NRT patches and to complete assessment instruments.<br><br>Provided with Nicoderm CQ™ patches weekly. 21 mg 10-week step-down regimen: 21 mg patch (Weeks 1–6), 14 mg patch (Weeks 7 and 8), and 7 mg patch (Weeks 9 and 10).<br><br>**Retention**		VRST (n=16) = 0.63, 1.2<br>S (p<.05)<br><br>2 months<br>NRTO (n=21) = 3.0, 3.6<br>VRST (n=15) = 0.74, 1.4<br>S (p<.05)<br><br>3months<br>NRTO (n=18) = 3.3, 3.9<br>VRST (n=13) = 1.0, 2.5<br>NS (p=.07)<br><br>6months<br>NRTO (n=11) = 7.4, 7.3<br>VRST (n=11) = 0.41, 0.86<br>S (p<.01)	

Authors<br>***Country***<br>***Years data collected***	***Setting***<br>***Sample characteristics (sample size, demographics)***	***Recruitment method***<br>***Eligibility criteria***	***Study design***<br>***Virtual reality intervention description***<br>***Control/comparators description***<br>***Retention at follow-up/end of treatment***	***Measures of health behaviours***	***Health behaviours outcomes***	***Secondary Outcomes***<br><br>***Use of support received***<br>***Satisfaction/acceptability of support received***<br>***Costs***
			53% (46/86) completed 10-week treatment.			
Goldenhersch et al. [27]<br>Argentina<br>2018	Residents of the City of Buenos Aires, Argentina (n=120)<br><br>**Sex**<br>48% female (57/120)<br>**Age**<br>43.25 years, 10.06<br>**Education**<br>High school 38% (45/120)<br><br>**Years since started smoking (mean, SD)**<br>19.15, 12.35<br><br>**Current cigarette consumption (cigarettes per day)**<br>10.77, 5.47<br><br>**At least 1 attempt to quit in the last year**<br>54% (65/120)	Unpaid advertisements were posted on the Mindcotine page on Facebook, and a 13-minute segment was aired on Argentine national public television. The social network advertisements linked invitations directly to a screening questionnaire on the Typeform platform.<br><br>**Eligibility criteria**<br>(1) be aged between 24 and 65 years; (2) consume a minimum of 5 cigarettes per day, with a score of 4 to 9 on the Contemplation Ladder; (3) be residents in the city of Buenos Aires; (4) own an Android mobile phone with gyroscope; (5) have a data plan or Wi-Fi access; and (6) have an interest in using VR as a method to quit smoking. Participants were excluded if they were diagnosed with a current	**Study design**<br>RCT<br><br>**Intervention**<br>N=60<br>The Mindcotine program utilises a smartphone app that consists of a 21-day treatment that includes 2 main activities each day, which become available after completing the previous day's activities.<br>The elements of the program were (1) practice sessions in formal mindfulness, (2) practice sessions in informal mindfulness using virtual reality mindful exposure therapy (VR-MET), (3) daily self-reports, (4) peer-to-peer support, and (5) Mindcotine support.<br><br>The Mindcotine app was downloaded onto smartphones and for the VR portion of the program the smartphone would be loaded into a headset for a fully immersive experience.<br><br>The VR aspect consisted of virtual environments that combined the awareness of the act of smoking and the recognition of craving from a perspective of acceptance and	**Smoking**<br>*Self-reported abstinence:* smoked in the last 24hrs<br><br>*Sustained abstinence self-report:* smoked in the last 90 days<br><br>*Cigarette consumption:* number of cigarettes smoked each day (self-report).	**Smoking outcomes**<br>*Self-report abstinence*:<br>At postintervention:<br>TG reported 23% (14/60) CG: 5% (3/60) S (χ 2 1=8.3; P=.004).<br><br>*Sustained abstinence at 90-day follow-up (between groups inconclusive due to drop-out):*<br>TG: 33% (20/60)<br>CG: 5% (3/60)<br>Statistical comparison not made as only 20% (12/60) of participants in CG completed 90-day follow-up.<br><br>*Cigarette consumption:*<br>Cigarettes per day over time (week 3 and post intervention):<br>TG vs CG<br>(F5,114=95.73; P<.001)<br><br>Mean number of cigarettes consumed each day at week 3 of	NA

Authors<br>***Country***<br>***Years data collected***	***Setting***<br>***Sample characteristics (sample size, demographics)***	***Recruitment method***<br>***Eligibility criteria***	***Study design***<br>***Virtual reality intervention description***<br>***Control/comparators description***<br>***Retention at follow-up/end of treatment***	***Measures of health behaviours***	***Health behaviours outcomes***	***Secondary Outcomes***<br><br>***Use of support received***<br>***Satisfaction/acceptability of support received***<br>***Costs***
		psychiatric disorder.	commitment.<br><br>Each of the virtual environments video recorded in locations in Mexico City. and the animated parts of the environments were created in Unreal Engine 4 (Epic Games). Each video was repeated over a period of 14 consecutive days.<br><br>**Control/comparator**<br>N=60<br>Received a self-help manual<br><br>**Retention**<br>97% (116/120) retention at 1-day follow up<br>57% (68/120) participants remained at 90-day follow up		intervention (mean, SD)<br>TG: 6.92, 5.26<br>CG: 9.03, 5.42<br>S (P=.03).<br><br>Postintervention (mean, SD)<br>TG: 5.07, 5.65<br>CG: 9.53, 0.56<br>S (P<.001).	
Karekla, Savvides, Gloster [28]<br>Cyprus<br>2014-2015	Three universities in Cyprus N=84<br><br>**Sex**<br>65% (55/84) female<br>**Age (mean, SD)**<br>22.44, 2.61<br>**Ethnicity**<br>90% Greek Cypriots<br><br>**Age of first cigarette (mean,**	Recruited from 3 universities in Cyprus via flyers posted in cafeterias and classroom announcements (during the academic year 2014–2015). Recruitment occurred in two rounds, one in the fall and one in the spring semester.<br><br>**Eligibility criteria**<br>Exclusion criteria included not being a daily smoker,	**Study design**<br>Randomized clinical trial<br><br>**Intervention**<br>N=49<br>Flexiquit program: A six-session avatar-led digitised acceptance and commitment therapy (ACT) program.<br><br>Six sessions, averaging 25 min each. The assessment and treatment were all completed online. Frequency of contact with the program was spaced out over 3–30 days; thus, the time it took to	**Smoking**<br>*Cessation status:*<br>Self-report 7-day point prevalence, “Did you smoke, even a puff, in the last 7 days?” (Yes/No).<br><br>*Number of cigarettes smoked*:<br>Per day	**Smoking outcomes**<br>*Cessation status:*<br>At post-treatment<br>Self-reported quit rates (7-day point-prevalence abstinence)<br>Flexiquit: 51.90%<br>Control: 14.30%<br>S (p = .005)<br><br>Note: 6-month comparisons not possible as CG entered treatment.	

Authors Country Years data collected	*Setting Sample characteristics (sample size, demographics)*	*Recruitment method Eligibility criteria*	*Study design Virtual reality intervention description Control/comparators description Retention at follow-up/end of treatment*	*Measures of health behaviours*	*Health behaviours outcomes*	*Secondary Outcomes*<br><br>*Use of support received Satisfaction/acceptability of support received Costs*
	**SD)**<br>16.36 years, 2.34<br>**Average cigarettes per day (mean, SD)**<br>9.41, SD = 6.93 (range = 1–27 cigarettes per day)<br>**Year of study Flexiquit**<br>First year=4.3%<br>Second year=21.4%<br>Third year=22.9%<br>Fourth year=24.3%<br>Postgraduate= 27.1%<br><br>**Waitlist control**<br>First year=11.4%<br>Second year=17.1%<br>Third year=11.4%<br>Fourth year=28.6%<br>Postgraduate= 31.4%	defined as smoking <1 cigarette per day over the past 30 days.	complete the entire program and post-assessment varied. To proceed to the next session the previous one had to be completed.<br><br>There were both constrained and unconstrained multiple-choice questions used for participants to communicate with the avatar. To build rapport, the avatar presented his/her own backstory and teachings regarding smoking. The engine was rules-based with several option paths with pre-recorded answers for each path. Thus, depending on the users' response to the questions posed by the avatar, there was a different continuation to the story, different information encouragement, skill, and so forth provided.<br><br>Virtual reward system of earning points for completing tasks and sessions, along with receiving congratulations, certificates, and a virtual graduation from the program celebration.<br><br>Participants were instructed/guided by the virtual avatar to watch instructional videos, interact in discussions, fill out questionnaires and practicing ACT based exercises/techniques.<br><br>**Control/comparator**		 *Number of cigarettes smoked per day*<br>Control group (mean, SD)<br>Pre: 8.39, 7,14<br>Post: 7.61, 7.26<br>Flexiquit group (mean, SD)<br>Pre: 8.48, 6.73<br>Post: 2.89, 4.01<br>At post-intervention<br>S ($p = .005$) $\eta2 = 0.14$<br><br>Time x Group interaction<br>S ($p = .003$), $\eta2 = 0.15$	

Authors<br>***Country***<br>***Years data collected***	***Setting***<br>***Sample characteristics (sample size, demographics)***	***Recruitment method***<br>***Eligibility criteria***	***Study design***<br>***Virtual reality intervention description***<br>***Control/comparators description***<br>***Retention at follow-up/end of treatment***	***Measures of health behaviours***	***Health behaviours outcomes***	***Secondary Outcomes***<br><br>***Use of support received***<br>***Satisfaction/acceptability of support received***<br>***Costs***
			N=35<br>Wait-list control<br><br>**Retention**<br>66% (55/84) retention at post-treatment after 6 sessions (time varied).			
Pericot-Valverde et al. [29]<br>Spain<br>NA	Participants were treatment-seeking smokers from the local community in, Barcelona and Oviedo, Spain (N=102)<br><br>**Sex (female)**<br>CBT group: 63.5% (33/52)<br>CBT+CET: 72% (36/50)<br>**Age (mean, SD)**<br>CBT group: 39.21 ± 12.97<br>CBT+CET: 39.68 ± 12.77<br>**Marital status (married)**<br>CBT group: 46.2% (24/52)<br>CBT+CET: 46.8% (23/50)<br>**Education (≥ high school)**	Participants were treatment-seeking smokers recruited via flyers and advertisements posted around the local community in, Barcelona and Oviedo, Spain.<br><br><br>**Eligibility criteria**<br>Being aged 18 or over, meeting the diagnostic criteria for nicotine dependence according to the Diagnostic and Statistical Manual of Mental Disorders assessed by the Structured Clinical Interview for DSM-IV (SCID-I), being interested in quitting smoking, and smoking 10 or more cigarettes per day for the prior 12 months.<br>Participants were excluded if they were	**Study design**<br>Two-site Randomized clinical trial<br><br>**Intervention**<br>N=50<br>*Cue exposure techniques (CET) + Cognitive Behavioral Treatment (CBT):* The CET component consisted of five sessions of individual exposure through virtual reality provided immediately before or after the CBT session. Virtual environments involved physical situations where people were smoking or offered cigarettes, having coffee, and drinking alcoholic beverages. They contained smoking paraphernalia, such as ashtrays, lighters, lit cigarettes, and cigarette vending machines. The virtual environments were as follows: being in a pub, having lunch at home, having breakfast at home, having coffee at a cafe, having lunch at a restaurant, waiting in the street, and watching TV at night.<br>During the exposure participants wore fitted VR eyewear and were able to	**Smoking**<br><br>**Smoking abstinence**<br>*Point-prevalence*: Having not smoked (not even a puff) for the past 24h at the end-of-treatment and for the past 7 days at follow-ups<br><br>*Continuous abstinence*: Quit smoking at the end-of-treatment and maintained abstinence throughout follow-ups.	**Smoking outcomes**<br>*Point-prevalence past 24hrs at end of treatment*<br>CBT+CET: 56%<br>CBT: 51.9%<br>NS<br>*Point-prevalence past 7-days at 1-month*<br>CBT+CET: 38%<br>CBT: 38.5%<br>NS<br>*Point-prevalence past 7-days at 6-months*<br>CBT+CET: 24%<br>CBT: 25%<br>NS<br>*Point-prevalence past 7-days at 12-months*<br>CBT+CET: 22%<br>CBT: 34.6%<br>NS<br><br>*Continuous abstinence (1-month)*<br>CBT+CET: 34%	**Adherence**<br>Attended all sessions<br>CBT+CET: 78%<br>CBT: 73.1%<br><br>Mean number of sessions<br>CBT+CET: 5.38 (SD = 1.38<br>CBT: 5.40 (SD = 1.33)

Authors<br>*Country*<br>*Years data collected*	*Setting*<br>*Sample characteristics (sample size, demographics)*	*Recruitment method*<br>*Eligibility criteria*	*Study design*<br>*Virtual reality intervention description*<br>*Control/comparators description*<br>*Retention at follow-up/end of treatment*	*Measures of health behaviours*	*Health behaviours outcomes*	*Secondary Outcomes*<br><br>*Use of support received*<br>*Satisfaction/acceptability of support received*<br>*Costs*
	CBT group: 63.5% (33/52)<br>CBT+CET: 74% (37/50)<br><br>**Cigarettes per day (mean, SD)**<br>CBT group: 19.4, 10.3<br>CBT+CET: 18.7, 9.2<br>**Years of regular smoking (mean, SD)**<br>CBT group: 21.0, 12.5<br>CBT+CET: 20.7, 10.7	diagnosed with a current severe psychiatric disorder (such as dementia or a psychotic disorder), if they met criteria for abuse of or dependence on a substance other than nicotine, or if they were currently involved in another smoking cessation treatment.	freely navigate and move through the virtual environment with a standard mouse device. Participants could engage in social interactions with avatars smoking or involved in activities related to smoking (e.g., drinking alcohol), engage in everyday life activities (e.g., eat, drink, walk on the street), and interact with executable objects within the virtual environment (e.g., opening pack of cigarettes, purchasing cigarettes). Participants were encouraged to carefully observe the situation in order to experience the highest level of cigarette craving. When the patient experienced a reduction in craving to baseline rating for that session or to baseline levels +15% when the exposure had exceeded 20 min, the screen automatically turned black and the exposure terminated.<br><br>**Control/comparator**<br>N=52<br>*CBT*: This consisted of a manualized intervention based on previous studies, implemented in group-based sessions of 5 to 6 individuals. Each session took approximately 60 min and occurred once a week over a <u>6-week period</u>. The treatment was highly structured and included: (a) information about tobacco, (b) a behavioural contract through	Verified abstinence by assessing expired CO levels	CBT: 37.2%<br>NS<br><br>*Continuous abstinence (6-months)*<br>CBT+CET: 18%<br>CBT: 19.2%<br>NS<br><br>*Continuous abstinence (12-months)*<br>CBT+CET: 16%<br>CBT: 17%<br>NS<br><br>*Participants who were unable to achieve abstinence at any point in time (the end-of-treatment, 1-, 6-, and 12-month follow-ups)*<br>CBT+CET: 40.0%<br>CBT: 44.2%<br>NS	

Authors Country Years data collected	*Setting Sample characteristics (sample size, demographics)*	*Recruitment method Eligibility criteria*	*Study design Virtual reality intervention description Control/comparators description Retention at follow-up/end of treatment*	*Measures of health behaviours*	*Health behaviours outcomes*	*Secondary Outcomes*<br><br>*Use of support received Satisfaction/acceptability of support received Costs*
			which the patients pledged to attend the sessions and quit smoking, (c) self-monitoring and graphical representation of cigarette consumption, (d) nicotine fading, (e) stimulus control, (f) strategies for controlling nicotine withdrawal symptoms, (g) physiological feedback (measured by expired CO), (h) training in alternative behaviours, (i) social reinforcement of objectives completion and abstinence, and (j) strategies to prevent relapse. Participants were instructed to quit between the fifth and the sixth treatment session.<br><br>**Retention**<br>83% (85/102) retained at 12-month follow-up.<br>81% (83/102) retained at 6-month follow-up.<br>84% (86/102) retained at 1-month follow-up.			
Woodruff et al. [30]<br>USA<br>2002-2004	Adolescent smokers recruited from high schools in San Diego County, USA (n=136)<br><br>**Sex**<br>46% (63/136) male	Teenage smokers recruited from 14 local high school via classroom presentations, lunch-hour sign-up tables, flyers, posters, school newspaper ads and articles, school-wide announcements, and school liaison referrals. Seven schools were in the	**Study design**<br>RCT<br><br>Participants in both conditions were surveyed at baseline, postintervention, 3-months post-intervention, and 12-months post-intervention.<br>**Intervention**<br>N=77<br>VR: An Internet-based, virtual reality	**Smoking**<br>*Self-report measures*: Most measures were standard items used elsewhere, particularly those from the National Youth Tobacco	**Smoking outcomes**<br>*Condition-by-time interactions*<br>*(4 time periods = baseline, postintervention, 3-months, and 12-months)*<br><br>**Past week abstinence**<br>VR: 14-35	NA

Authors Country Years data collected	***Setting Sample characteristics (sample size, demographics)***	***Recruitment method Eligibility criteria***	***Study design Virtual reality intervention description Control/comparators description Retention at follow-up/end of treatment***	***Measures of health behaviours***	***Health behaviours outcomes***	***Secondary Outcomes***<br><br>***Use of support received Satisfaction/acceptability of support received Costs***
	**Age (mean, range)**<br>16, 14-19<br>**Ethnicity**<br>51% (69/136) Hispanic<br>**Continuation/alternative high schools**<br>41% (56/136)	control arm, and seven in the intervention arm.<br><br>**Eligibility criteria**<br>Student must have smoked at least one cigarette within the past 30 days.	world combined with motivational interviewing conducted by a smoking cessation counsellor.<br><br>The intervention consisted of seven 45-minute virtual world sessions over a 7-week period.<br>Sessions covered motivation to quit, self-efficacy for quitting sharing experiences of quitting, barriers and strategies to overcome them, goal setting, relapse, cues and influences, and education.<br>The virtual environment in which participants can see each other as 3-dimensional figures (i.e., avatars) on their computer screens, move around in the "world," and have real-time discussions with each other.<br>A sky mall was chosen as the setting because malls frequently serve as meeting places for teens. Within the sky mall, there were various virtual storefronts that supported the content of the counselling.<br>Different virtual settings included:<br>Pathology lab – displaying pictures of diseased organs and premature aging.<br>Art gallery – showing tobacco advertising and anti-tobacco art,<br>Billboards displaying topics such as 'Dealing with Cravings".<br>Graveyards – celebrities who have died from smoking-related diseases.	Survey/American Legacy Foundation, and the California Youth Tobacco Survey.<br>Questions pertained to:<br>Past week abstinence<br><br>Number of days smoked in the past 7 days<br><br>Number of cigarettes smoked per day	CG: 29-22<br>Pre-post: -1.401<br>S ($p < 0.01$)<br>4 time periods: -.106<br>NS<br><br>**Number of cigarettes smoked per day/past week**<br>VR: 3-2.2<br>CG: 2.3-2.5<br>Pre-post: -.989<br>S ($p<0.01$)<br>4 time periods: -.155<br>NS<br><br>**Number of days smoked in the past 7 days**<br>VR: 4.6-2.1<br>CG: 3.5-3.4<br>Pre-post: -1.753<br>S ($p<0.01$)<br>4 time periods: -.249<br>NS<br><br>Note: at 12-month follow up groups were nearly identical for above measures.	

Authors Country Years data collected	*Setting Sample characteristics (sample size, demographics)*	*Recruitment method Eligibility criteria*	*Study design Virtual reality intervention description Control/comparators description Retention at follow-up/end of treatment*	*Measures of health behaviours*	*Health behaviours outcomes*	*Secondary Outcomes*<br><br>*Use of support received Satisfaction/acceptability of support received Costs*
			These settings acted as prompts for discussion and education.<br><br>**Control/comparator**<br>N=59<br>No intervention control group (CG): A measurement-only condition asked only to complete the 4 online surveys.<br><br>**Retention**<br>75% (102/136) retained at end of study (7-weeks).<br>73% (99/136) retained at 12-month follow-up.			

Physical Activity

Basha, Aboelnour, Aly, Kamel [31]<br>Cairo, Egypt<br>2016-2019	Children from the burn unit of Teaching Hospitals, Cairo, Egypt (n=43)<br><br>Baseline data excluded dropouts (n=40)<br>**Sex**<br>65% (26/40) male<br>***10-16 Age* (mean, SD)**	NA<br><br>**Eligibility criteria**<br>Criteria for inclusion were burns on 40% to 60% of the total body surface area (TBSA) and capable of walking without an assistant. Children with issues such as disorders in the visual or vestibular system, metabolic disorders, amputation,	**Study design**<br>Monocentric RCT<br><br>**Intervention**<br>N=21<br>**Xbox Kinect training group**: interactive rehabilitation programme for 40-min sessions in addition to SPTP session.<br>- weeks 1–4: 2 repeats, with 5 min in basic free play mode. Rest for 60 sec between repeats;<br>- weeks 5–8: 2 repeats, with 5	**Physical activity**<br>*Cardiopulmonary fitness*: VO2peak measured using a modified Bruce protocol treadmill test. 3-min stages that gradually increased in speed and	**Physical activity outcomes**<br>*Vo2 Peak at 12-weeks (mean, SD)*<br>XG: 30.1, 2.22<br>CG: 26.85, 1.42<br>S (p<0.001)	**Adherence**<br>Median treatment adherence rates were **96%** (Interquartile range [IQR] 91–100) in the Xbox group and **93%** (IQR 89–97) in the control group, with no difference between groups (P = 0.27).

Authors Country Years data collected	*Setting Sample characteristics (sample size, demographics)*	*Recruitment method Eligibility criteria*	*Study design Virtual reality intervention description Control/comparators description Retention at follow-up/end of treatment*	*Measures of health behaviours*	*Health behaviours outcomes*	*Secondary Outcomes*<br><br>*Use of support received Satisfaction/acceptability of support received Costs*
	XG: 12.7, 1.56<br>CG: 13.3, 1.29<br>***Height (cm)* (mean, SD)**<br>XG: 153.4, 4.96<br>CG: 154.8, 5.91<br>***Weight (kg)* (mean, SD)**<br>XG: 46.3, 6.04<br>CG: 47.1, 5.46	neuropathy, history of neurological disorders, disequilibrium, deformity of the upper and lower limbs and involvement in another study were excluded.	min in intermediate free play mode. Rest for 60 sec between repeats;<br>- weeks 9–12: 2 repeats, with 5 min in advanced free play mode. Rest for 60 sec between repeats.<br><br>Games were displayed on a 50-inch LG screen. Xbox training consisted of 4 games (Rally Ball, Reflex Ridge, River Rush and 20000 Leaks). Children played each game for 10 min<br><br>**Control/Comparator**<br>N=22<br>All children in control and intervention groups were engaged in a standard physical therapy protocol (SPTP) conducted in 60-min sessions for 3 days per week for 12 weeks.<br>SPTP included splinting, walking every day, joint range motion exercises, muscle stretching procedures, scar management, and ADL training.<br><br>**Retention**<br>93% (40/43) retained at 12-weeks	incline-expired gases measured until volitional exhaustion.<br>Face mask calibrated expired gas analysis system		**Satisfaction/ acceptability**<br>Xbox training group reported significant enjoyment as compared with the control group (P < 0.001). Enjoyment scores were higher in the Xbox training than control group (mean [SD] 31.7 [2.1] vs. 19.05 [2.6])
Chuang, Sung, Chang, Wang [32]<br>Taiwan	Veterans Affairs Medical Center Taipei in Taiwan (n=24)	Subjects were prospectively recruited from the cardiovascular surgery department at the	**Study design**<br>Clinical RCT<br><br>**Intervention**	**Physical activity**<br>***Exercise intensity***	**Physical activity outcomes**<br>*Treadmill grades and speeds*:	NA

Authors Country Years data collected	*Setting Sample characteristics (sample size, demographics)*	*Recruitment method Eligibility criteria*	*Study design Virtual reality intervention description Control/comparators description Retention at follow-up/end of treatment*	*Measures of health behaviours*	*Health behaviours outcomes*	*Secondary Outcomes*<br><br>*Use of support received Satisfaction/acceptability of support received Costs*
2004	Demographics reported (N=20)<br>**Sex**<br>100% male<br>**Age (mean, SD)**<br>Non-VR group: 63.7, 10.03<br>VR group: 65.70, 14.48	Veterans Affairs Medical Center Taipei, Taipei, Taiwan,<br><br>**Eligibility criteria**<br>Had received coronary artery bypass grafting (CABG).<br>Included if they qualified for the supervised outpatient cardiac rehabilitation programs.	N=12<br>Telepresence Cardiac Rehabilitation Program, in which users are physically active in and interactive with an imaginary 3D setting. The system permits speed alteration and treadmill incline adjustments in conjunction with scenery changes.<br>The VR scenes show a 3D-constructed "virtual runner" model. The scenes were fused into standard 2-dimensional background descriptions.<br>The images were projected from behind the viewer through 3 projectors connected with computers. Within this field of view, the eyes can register the objects surrounding the viewer. The virtual terrain in the study consisted of a 5-km-long straight (or curved) stretch of road, grass, and trees with a mountain background. Once the treadmill was attached to the PC system, the rate of the subject's movement. The VR programs also offered immediate biofeedback on the subject's condition.<br><br>**Control/comparator**<br>N=12<br>The comparator subjects performed treadmill walking at measured speeds and grades without VR.<br><br>**Retention**	*Treadmill grades and speeds*: highest speeds reached(mph) based on participants maximum exercise tolerance.	highest speed (mph) (X, SD)<br>VR: 4.64, 1.4<br>Non-VR: 3.70 ,0.81<br>S (P=.037)	

Authors Country Years data collected	*Setting Sample characteristics (sample size, demographics)*	*Recruitment method Eligibility criteria*	*Study design Virtual reality intervention description Control/comparators description Retention at follow-up/end of treatment*	*Measures of health behaviours*	*Health behaviours outcomes*	*Secondary Outcomes*<br><br>*Use of support received Satisfaction/acceptability of support received Costs*
			83% (20/24) retention at the end of the 3-month intervention.			
Chuang, Sung, Lin [33]<br>Taiwan<br>2001-2003	Veterans Affairs Medical Center Taipei in Taiwan (n=49)<br><br>Baseline reported (N=32)<br>**Sex:** 88% (28/32) male<br>**Age (mean, SD)**<br>Non-VR group: 68.67, 12.32<br>VR group: 64.41, 7.66	Patients were consecutively referred by their physicians to the cardiopulmonary laboratory of the Department of Physical Medicine and Rehabilitation of this institute (Veterans Affairs Medical Center Taipei in Taiwan) after submitting to coronary artery bypass grafting (CABG) surgery between 2001 and 2003.<br><br>**Eligibility criteria**<br>Undergoing bypass surgery.	**Study design**<br>Prospective randomised controlled study design<br><br>All of the subjects were encouraged to perform submaximal endurance exercises twice weekly, for about 3 months in the hospital, at a level that corresponded to (1) 70% to 80% maximal heart rate, or (2) 60% to 75% VO2max, or (3) a perceived exertion rating of 11 to 15 on the Borg scale (beta-blocker user). The physiotherapist adjusted the treadmill speed and grade every 5 minutes and sessions lasted for up to 30 minutes, or until the patient had achieved the appropriate level of exertion.<br><br>**Intervention**<br>N=NA<br>Telepresence Cardiac Rehabilitation Program, in which users are physically active in and interactive with an imaginary 3D setting. The system permits speed alteration and treadmill incline adjustments in conjunction with scenery changes.<br>The VR scenes show a 3D-constructed “virtual runner” model. The scenes were fused into standard 2-dimensional	**Physical activity**<br>*Heart rate:* electrocardiography machine (Target between 70% and 80% HRmax)<br><br>Peak Vo2: the speed and incline of the treadmill at 75% Vo2peak during the exercise testing session.<br><br>Peak Metabolic equivalents (METS)	**Physical activity outcomes**<br>(1) significant gain in VO2peak and peak MET value accrued to those who participated in simulation-based aerobic training compared with those in non-VR settings; (2) at the follow-up exercise tests, statistical significances were attained between groups for VO2peak, peak MET, and amount of VO2 at anaerobic threshold.<br><br>***Mean. SD at Follow-up (FU)***<br>*Maximal HR*<br>Non-VR group (FU): 128.47, 15.85<br>VR group (FU): 135.24 12.40<br>*Significance (P)*<br>FU Between: .186<br>Within: .310<br><br>*Peak Vo2*	NA

Authors Country Years data collected	*Setting Sample characteristics (sample size, demographics)*	*Recruitment method Eligibility criteria*	*Study design Virtual reality intervention description Control/comparators description Retention at follow-up/end of treatment*	*Measures of health behaviours*	*Health behaviours outcomes*	*Secondary Outcomes*<br><br>*Use of support received Satisfaction/acceptability of support received Costs*
			background descriptions.<br>The images were projected from behind the viewer through 3 projectors connected with computers. Within this field of view, the eyes can register the objects surrounding the viewer. The virtual terrain in the study consisted of a 5-km-long straight (or curved) stretch of road, grass, and trees with a mountain background. Once the treadmill was attached to the PC system, the rate of the subject's movement. The VR programs also offered immediate biofeedback on the subject's condition.<br><br>**Control/comparator**<br>N=NA<br>The comparator subjects performed treadmill walking at measured speeds and grades without VR.<br><br>**Retention**<br>65% (32/49) retention at the end of the 3-month intervention.		Non-VR group (FU): 16.84, 4.64<br>VR group (FU): 22.47, 4.48<br>*Significance (P)*<br>FU Between: .002*<br>Within: .037*<br><br>*Peak METS (mean, SD)*<br>Non-VR group (FU): 4.81, 1.33<br>VR group (FU): 6.42, 1.28<br>*Significance (P)*<br>FU Between: .002*<br>Within: .037*	
Friederichs et al. [34]<br>Netherlands<br>2012	Dutch residents who occasionally volunteer in Web-based research (n=958)<br><br>**Age (mean)**<br>42.9	The participants were recruited through an Internet panel of Dutch residents who occasionally volunteer in Web-based research.<br><br>**Eligibility criteria**	**Study design**<br>Three-arm RCT<br><br>Measurements were taken using Web-based<br>questionnaires at baseline, directly after the intervention (follow-up 1 – 4 weeks) and 1 month post intervention (follow-	**Physical Activity**<br>The number of weekly days with at least 30 minutes of moderate PA was measured	**Physical Activity outcomes**<br>*Days per week engaging in moderately physical activity for at least 30 minutes*:<br>At 1 month, participants were on	**Satisfaction/acceptability**<br>Process evaluation results were positive (ie, entertainment 5.16/7; trustworthiness

Authors Country Years data collected	***Setting Sample characteristics (sample size, demographics)***	***Recruitment method Eligibility criteria***	***Study design Virtual reality intervention description Control/comparators description Retention at follow-up/end of treatment***	***Measures of health behaviours***	***Health behaviours outcomes***	***Secondary Outcomes***<br><br>***Use of support received Satisfaction/acceptability of support received Costs***
	**Sex**<br>60.4% (578/958) female	Impairments that severely impede PA participation (participants were asked whether they were unable to be physically active), not speaking and/or writing Dutch, and not having Internet access.	up 2).<br><br>**Intervention**<br>N=NA<br>*Web-based PA intervention based on MI with an avatar (AVATAR)*: Avatar (male or female) positioned behind a desk in a small office. Questions communicated through text balloons. Avatar displays speech movements, social dialogue and non-verbal expressions.<br><br>**Control/Comparator**<br>N=NA<br>Content-identical intervention without an avatar (TEXT):<br>In both interventions current PA behaviour, perceived importance of PA, benefits of PA and participant confidence in successfully becoming more PA.<br><br>N=NA<br>Control: received no intervention.<br><br>**Retention**<br>52% (500/958) participants (AVATAR 162; TEXT 146; CONTROL 192) remained at 1-month follow-up<br><br>Dropout analyses showed that participants younger than age 46 were more likely to drop out at 1 month (OR 1.95, 95% CI 1.51-2.53)	with a self-reported single item of the Dutch Short Questionnaire to Assess Health Enhancing Physical Activity (SQUASH)	average moderately physically active for an average of 4.4 (SD 1.8) days per week for at least 30 minutes per day.<br>AVATAR: mean **4.6** [SD 1.6]; TEXT: mean **4.7** [SD 1.8]; CONTROL: mean **4.0** [SD 1.9]).<br><br>AVATAR vs CONTROL=.39, S (P=.011)<br>TEXT vs CONTROL =.44, S (P=.006)<br>AVATAR vs TEXT = NS	5.15/7; overall appreciation score 7.14/10). No significant differences were found between the intervention conditions regarding these variables.

Authors Country Years data collected	***Setting Sample characteristics (sample size, demographics)***	***Recruitment method Eligibility criteria***	***Study design Virtual reality intervention description Control/comparators description Retention at follow-up/end of treatment***	***Measures of health behaviours***	***Health behaviours outcomes***	***Secondary Outcomes***<br><br>***Use of support received Satisfaction/acceptability of support received Costs***
Gulsen et al. [35]<br>Turkey<br>2018-2019	Gazi University Hospital, Department of Algology, Turkey (n=20).<br><br>Baseline data available (N=16)<br>**Sex**<br>100% (16/16) female<br>**Age (Median, IQR)**<br>Exercise group: 38.5, 29.5-50.0)<br>IVR group: 46.5, 36.5-49.5<br>**BMI (Median, IQR)**<br>Exercise group: 22.85 (20.52–28.56)<br>IVR group: 26.81 (23.74–30.69)<br>**Education Level**<br>Exercise group: 37.5% (3/8) Primary education<br>IVR group: 50% (4/8) Primary education	Participants were recruited from Gazi University Hospital, Department of Algology.<br><br>**Eligibility criteria**<br>(a) diagnosed with Fibromyalgia (FM) in accordance with the American College of Rheumatology criteria by a specialist physician, (b) accepting to participate in the study, (c) being between the age of 18 and 65 years, (d) not having a cardiovascular, pulmonary, hormonal, or orthopedic disease, (e) not having an inability to prevent doing exercise, (f) being able to communicate effectively with the researchers. Exclusion criteria were: (a) having any vision, hearing, perception, or sensation problem that may affect research results, (b) having additional rheumatological disease (rheumatoid arthritis,	**Study design**<br>Singly blinded randomised controlled clinical trial<br><br>**Intervention**<br>N=10<br>Exercise+IVR group had 20 minutes of IVR treatment in addition to the same protocol as the Exercise only group (see below).<br><br>Fully immersive virtual reality (IVR) combined with exercise group: The systems used were:<br>(1) Computer: Consisted of system unit, monitor, keyboard, mouse, and speaker.<br>(2) Infrared Camera: Xbox Kinect sensor was used to detect body movements.<br>(3) Head-mounted display: Oculus head-mounted display was used.<br><br>Two games were used for IVR training which were developed with the help of RAGU (Augmented Reality Applications in Rehabilitation) System and which aimed to improve balance and mobility. The first was a 10-minute football game in which the patient was asked to counter the balls from different heights by using their hands and feet. The second was a 10-minute dungeon game, the patient was asked to avoid	**Physical activity**<br>*Level of physical activity:* Turkish version of long form of the International Physical Activity Questionnaire (IPAQ). Examines frequency, intensity, and total time of physical activity in different situations such as; work, transportation, housework-house care family care, recreation-sport-free time, and total time of sitting in last week. Higher scores indicate higher level of activity	**Physical activity outcomes**<br>*Level of physical activity:*<br>Comparison of changes between groups (baseline-8th week).<br>**(Median score, IQR)**<br>Exercise group: 528.50 (353.62; 929.00)<br>Exercise + IVR group: 1797.25 (1408.00; 4093.00)<br>S ($p<0.001$)<br><br>Within group differences between baseline and after the 8-week.<br>***Median score, IQR***<br>*Exercise group*<br>Before: 919.00 (102.37–1298.25)<br>After: 1430.25 (942.00–1778.25)<br>S ($p<0.012$)<br><br>*Exercise + IVR group:*<br>Before: 1095.75 (810.75–1696.50)<br>After: 2742.00 (2298.00–5735.25)	NA

Authors Country Years data collected	*Setting Sample characteristics (sample size, demographics)*	*Recruitment method Eligibility criteria*	*Study design Virtual reality intervention description Control/comparators description Retention at follow-up/end of treatment*	*Measures of health behaviours*	*Health behaviours outcomes*	*Secondary Outcomes*<br><br>*Use of support received Satisfaction/acceptability of support received Costs*
	**Participation of Regular Physical Activity**<br>100% No	osteoarthritis, etc.), (c) using medication other than the prescribed pharmacologic agents for FM symptoms, (d) have been using opioids for long-term. Patients were removed from the study if they had injection for tender points during intervention period.	guillotines by tilting the trunk to the right, left, forward and backward without moving his/ her feet and logs by standing on single leg or jump with both legs. The level of difficulty was increased according to the patient's performance in both games. Balls speed was increased gradually in football game, and direction of ball randomly was adjusted to patient's foot or hand in order to increase the difficulty. Coming order of guillotines and logs were also randomly adjusted for the difficulty. Patients could see their scores at the end of the games. To increase the participation, they were encouraged to make better scores than previous session. A harness system was used to ensure patients safety.<br><br>**Control/comparator**<br>N=10<br>*Exercise group*:<br>The exercise group had a combined exercise training consisting of 30 minutes of aerobic training and 30 minutes of Pilates which included strengthening and flexibility exercises.<br><br>**Retention**<br>80% (16/20) retention at 8-weeks.		S (p<0.012)	
Karssemeijer et al. [36]	Conducted in community centres	Participants were approached via the	**Study design**<br>Three-arm RCT	**Physical activity:**	**Physical activity outcomes**	**Adherence rates:**<br>EG: 87.3%

Authors<br>*Country*<br>*Years data collected*	*Setting*<br>*Sample characteristics (sample size, demographics)*	*Recruitment method*<br>*Eligibility criteria*	*Study design*<br>*Virtual reality intervention description*<br>*Control/comparators description*<br>*Retention at follow-up/end of treatment*	*Measures of health behaviours*	*Health behaviours outcomes*	*Secondary Outcomes*<br><br>*Use of support received*<br>*Satisfaction/acceptability of support received*<br>*Costs*
Netherlands<br>2016-2017	in Nijmegen, the Netherlands<br>(n=115)<br><br>**Age (mean, SD)**<br>Exergame group = 79, 6.9<br>Aerobic group = 80.9, 6.1<br>Control group = 79.8, 6.5<br>**Sex**<br>54% (62/115) Male<br>**Living situation**<br>56% (64/115) independent, with others<br>Exergame group = 50% (19/38) independent, with others<br>Aerobic group = 55% (21/38) independent, with others<br>Control group = 62% (24/39) independent, with others	memory clinic of Radboudumc Alzheimer Center, day care centres for adults with cognitive disorders, advertisement in local newspapers, and word of mouth.<br><br>**Eligibility criteria**<br>As reported in a preceding paper Karssemeijer et al., 2019, “The quest for synergy between physical exercise and cognitive stimulation via exergaming in people with dementia: A randomized controlled trial.”<br><br>People with a clinically confirmed diagnosis of dementia (vascular, Alzheimer’s, or mixed type), a Mini-Mental State Examination score of ≥ 17, and aged 60 years or older; if using anti-dementia medication, a stable dose for at least 3 months before the start of the trial; and being capable of giving informed consent.	All participants received 3 training sessions per week for 12 weeks.<br>**Intervention**<br>N=38<br>Exergame training group (EG): enrolled in a cognitive-aerobic bicycle training on a stationary bike connected to a video screen. The aerobic training component consisted of cycling for 30 to 50 minutes per session and aimed to achieve an intensity of 65% to 75% of heart rate reserve after 12 weeks of training. The cognitive training component consisted of following a route through a digital environment (often a city familiar to the participant, yielding pleasant memories) while performing cognitive tasks targeting response inhibition, task switching, and processing speed. These cognitive tasks were incorporated in the cycling routes that were shown on the video screen. There were 7 different cognitive training levels, and the difficulty of the cognitive tasks increased per training level to ensure that the training remained cognitively challenging. At the end of each training session, participants were provided with feedback on their scores. When the participants had a response time of less than 5 seconds and an error rate of less than 5% on the cognitive activity, they	Physical Activity Scale for the Elderly (PASE) Score: higher scores indicate higher levels of physical activity.<br><br>Resting heart rate and heart rate during training<br><br>Training intensity: % of maximal heart rate	No significant differences were found between the groups on level of physical activity (PASE)<br>**(mean, SD) (baseline=T0; 12-week=T1; 24-week=FU)**<br>**EG**:<br>T0 - 66.5(55.6)<br>T1 - 78.6 (60.l)<br>FU - 65.5 (55.5)<br><br>**AG**:<br>T0 - 74.2 (61.2)<br>T1 - 89.4 (78.5)<br>FU - 72.4 (51.4)<br><br>**CG**:<br>T0 - 52.9 (36.5)<br>T1 - 55.1 (44.2)<br>FU - 54.2 (45.1)<br><br>Between group NS<br><br>**Heart rate difference (mean, SD)**<br>EG: 26.1 (15.1)<br>AG: 26.0 (13.8)<br>CG: NA<br>NS	(SD=13.6)<br>AG: 81.1% (SD=13.7)<br>CG: 85.4% (SD=12.9)<br>F(2, 112)=2.86, P=.06*<br>*A trend was found toward higher adherence in the exergame group compared to the aerobic group (mean difference: 6.85, 95% CI: 0.09, 13.79; P = .05).<br><br>**Satisfaction**<br>Rating of training sessions (scale 1-5). Median (interquartile range)<br>EG: 5.0 (4-5)<br>AG: 5.0 (4-5)<br>CG: 5.0 (4-5)<br>$X^2(2) = 0.43$, P = .81

Authors<br>*Country*<br>*Years data collected*	*Setting*<br>*Sample characteristics (sample size, demographics)*	*Recruitment method*<br>*Eligibility criteria*	*Study design*<br>*Virtual reality intervention description*<br>*Control/comparators description*<br>*Retention at follow-up/end of treatment*	*Measures of health behaviours*	*Health behaviours outcomes*	*Secondary Outcomes*<br><br>*Use of support received*<br>*Satisfaction/acceptability of support received*<br>*Costs*
		Exclusion criteria were: co-morbidity that limited exercising, including severe cardiovascular, musculoskeletal or neurological disease; diagnosis of a depression, bipolar disorder or psychotic disorder at the moment of inclusion; current drug or alcohol dependency; exercising more than five times per week for at least 30 min at a moderate intensity; wheelchair bound; and severe hearing or visual problems that could not be corrected with the use of hearing aids/glasses.	could proceed to the next level<br><br>**Control/Comparator**<br>N=38<br>Aerobic training group (AG): cycling training on a stationary bike. This bike was not connected to a video screen. The aerobic training itself was identical to the training described above.<br><br>N=39<br>Active control group (CG): received a 30-minute-per-session training that consisted of relaxation and flexibility exercises.<br><br>All participants received 3 training sessions per week for 12 weeks.<br><br>**Retention**<br>80% (92/115) retained at final 24-week follow-up from randomization. 14 of which dropped out during 12-week intervention, and 9 at 24-week follow-up.		**Training intensity (mean, SD)**<br>EG: 41.8 (13.3)<br>AG: 43.5 (18.2)<br>CG: NA<br>NS	
Navarro et al. [37]<br>Spain<br>NA	Overweight and obese women – nutrition clinics and gyms (n=48)<br><br>**Sex**<br>100% women	Participants were recruited in nutrition clinics and gyms. Because participants had to show low activity levels, the gyms only contacted women who had dropped	**Study design**<br>RCT<br><br>First completed online questionnaire, followed by online intervention ("Motivation for change", and "Move it" – motivation, goal setting, and edu)	**Physical activity**<br>*International physical activity questionnaire (IPAQ):*<br>Through 31	**Physical activity outcomes**<br>IPAQ:<br>Regarding the ANOVA results, there was a main effect of time on PA levels F (1, 39) =	NA

Authors<br>Country<br>Years data collected	***Setting<br>Sample characteristics (sample size, demographics)***	***Recruitment method<br>Eligibility criteria***	***Study design<br>Virtual reality intervention description<br>Control/comparators description<br>Retention at follow-up/end of treatment***	***Measures of health behaviours***	***Health behaviours outcomes***	***Secondary Outcomes<br><br>Use of support received<br>Satisfaction/acceptability of support received<br>Costs***
	**Age (mean, SD)**<br>31.9, 11.7<br><br>**BMI (mean, SD)**<br>28.7, 3.1	out. Flyers and in-person presentations were used to publicize the study.<br><br>**Eligibility criteria**<br>Being a woman from 18–64 years old; being overweight (BMI > 25); having high body dissatisfaction (Body Schema Questionnaire—BSQ— > 80); being physically inactive; and not having any physical condition that could keep them from practicing PA.	participants followed for a week to increase PA. Then invited to laboratory, where virtual PA task applied for 10-minutes. The VR scenario consisted of a 3D graphical environment representing a park where an avatar runs. Avatars' characteristics varied depending on the experimental condition (IAC and RAC). In the IAC and RAC conditions, the participant's face was tracked by the Kinect. All participants ran in place in a room, and their movements were captured by a Kinect and projected on a 150 × 150 cm screen. During the PA task, participants could see the time and distance they had run on the screen.<br>**Intervention**<br>N=15<br>"Ideal avatar" (IAC: participants are represented by avatars with ideal body dimensions):<br>IAC participants were asked to create an avatar with their ideal body dimensions and their own face. They were shown a default avatar and were able to change its body dimensions. Then, they performed a running task for 4 min in a VR scenario where they were represented by this avatar. The VR task performance was video-recorded, and participants received this video on their mobile phones and were asked to watch it every day of the week.	items, this questionnaire collects data on PA performed in the past 7 days. It identifies the frequency and duration of moderate and vigorous leisure, transportation, and occupational PA, walking PA, and inactivity during the past week.<br><br>Note: reported test-retest reliability correlations of 0.81 and validity correlations with accelerometers of 0.33<br><br>PA Goals: Walking or	15.82, p = 0.000, η = 0.29. All participants showed higher PA levels after the intervention. However, the interaction between time and condition was not significant F (1, 39) = 0.05, p = 0.949, η = 0.00.<br><br>*Pre*<br>NAC: 2499.81 (2231.45)<br>RAC: 1902.33 (971.67)<br>IAC: 2552.98 (1927.08)<br>Total: 2318.37 (1773.36) P=0.571<br><br>Post<br>NAC: 3884.92 (2671.75)<br>RAC: 3065.23 (1924.58)<br>IAC: 3733.87 (2428.44)<br>P=0.949<br><br>Within-group Effect Size, d [95% CI] Pre-post Intervention<br>NAC: −0.58 [−1.14, −0.03]<br>RAC: −1.13 [−1.81,	

Authors Country Years data collected	*Setting Sample characteristics (sample size, demographics)*	*Recruitment method Eligibility criteria*	*Study design Virtual reality intervention description Control/comparators description Retention at follow-up/end of treatment*	*Measures of health behaviours*	*Health behaviours outcomes*	*Secondary Outcomes*<br><br>*Use of support received Satisfaction/acceptability of support received Costs*
			N=14<br>"Real avatar" (RAC: participants are represented by avatars with participants' current body dimensions): RAC participants received the same instructions, but they were asked to change the avatar (with their face) to fit their real body dimensions.<br><br>**Control/comparator**<br>N=19<br>"Non avatar" (NAC: participants are not represented by avatars):<br>NAC participants were asked to perform the PA task in the VR scenario for 4 min, but participants were not represented by an avatar. They ran in front of a fixed image corresponding to the VRE. They did not receive any video-recordings.<br><br>**Retention**<br>88% (42/48) retained at the end of the three-week program	running three times a week.	−0.44]<br>IAC: −0.58 [−1.13, −0.02]<br>It is important to highlight the changes observed in the RAC condition. According to standardized effect sizes (Cohen's d), this group obtained a large effect size (>0.80) for their change in PA levels, and they increased their weekly practice the most.<br><br>*PA Goals (Walking or Running):*<br>PA goals (walking or running three times a week). No significant effects were found on the achievement of the PA goal F (2, 41) = 0.36, p = 0.702, η = 0.02.	
Ruiz et al. [38]<br>USA<br>NA	Miami VAHS primary care clinics (n=30)<br><br>Analysed at baseline (n=28)<br>**Sex**	NA<br><br>**Eligibility criteria**<br>Cognitively intact (normal Mini-Cog).<br>Non-depressed veterans.<br>>50 years old	**Study design**<br>RCT<br><br>10 minutes presentation on basic principles about physical activity + set of instruction on how to perform different exercises.	**Physical activity**<br>Daily energy expenditure: Accelerometer measuring Kcals/day.	**Physical activity outcomes**<br>*Between groups mean difference in kcal/day expenditure gains during physical activity (mean, SD)*	NA

Authors Country Years data collected	*Setting Sample characteristics (sample size, demographics)*	*Recruitment method Eligibility criteria*	*Study design Virtual reality intervention description Control/comparators description Retention at follow-up/end of treatment*	*Measures of health behaviours*	*Health behaviours outcomes*	*Secondary Outcomes*<br><br>*Use of support received Satisfaction/acceptability of support received Costs*
	96% (27/28) male<br>**Age (mean, SD)**<br>62, 6<br>**BMI (mean, SD)**<br>31, 4<br>**Weight (kg) (mean, SD)**<br>220, 34<br>**Tried to lose weight before**<br>71% (20/28)<br>**Confidence to change eating or physical activity habits (10-point Likert scale) (mean, SD)**<br>7.64, 2.32	Overweight or obese BMI >25kg/m²<br>Sedentary lifestyle (exercising less than 90 minutes/week in the previous two-week period)	Participants took part in one 10-minutes VR session per visit (weeks 0, 2 and 4) on a pc at the research clinic. The virtual 3D environment consisted of an exercise room and photorealistic avatars. Each session was 45 minutes long.<br>On weeks 1, 3, 6 and 8 data collection questionnaires took place over the phone.<br>**Intervention**<br>N=9<br>Virtual representation of the Self exercising condition (VRS): the 12 animated exercise routines had a virtual avatar with a head that resembled the participants.<br><br>**Control/comparator**<br>N=10<br>Virtual representation of other person exercising (VRO): the avatar's head featured an unknown person's head of the same sex, skin colour and approximate age.<br><br>N=9<br>Control condition: Participants observed static graphics depicting the different physical activity routines instead of an animated avatar.<br><br>**Retention**		VRS: 29, 23<br>VRO: 147, 160<br>CG: 57, 100<br>NS<br><br>It is important to note all the groups have large effect size of the expenditure gain after the intervention (r>0.50)	

Authors Country Years data collected	*Setting Sample characteristics (sample size, demographics)*	*Recruitment method Eligibility criteria*	*Study design Virtual reality intervention description Control/comparators description Retention at follow-up/end of treatment*	*Measures of health behaviours*	*Health behaviours outcomes*	*Secondary Outcomes<br><br>Use of support received Satisfaction/acceptability of support received Costs*
			70% (21/30) retained at post-intervention (week-8 phone follow-up of the study – 4 weeks post face to face).			
Thomas et al. [39]<br>UK<br>2013-2014	Patients with Multiple Sclerosis (MS) at Poole Hospital, Poole, Dorset, UK (n=30)<br><br>**Sex**<br>90% (27/30) female<br>**Age (mean, SD)**<br>49.3, 8.7<br>**Tried active gaming**<br>60% (18/30) new to active gaming<br>**Ethnicity**<br>90% (27/30) White English<br>**Years since diagnosis**<br>37% (11/30) 1-5 years<br>**Employment status**<br>37% (11/30) not in paid employment<br>**Marital status**<br>70% (21/30)	Recruited via multidisciplinary team members of an MS Service in a secondary care setting (Poole Hospital NHS Foundation Trust).<br>Patients were either given or sent an invitation letter.<br><br>**Eligibility criteria**<br>(i) a clinically definite diagnosis of MS; (ii) aged 18 years or above; (iii) satisfied a risk assessment; (iv) relatively physically inactive (active for a period of 30min or more on fewer than 5 days per week; (vi) having a suitable television at home.<br><br>Excluded if:<br>(i) Adapted Patient Determined Disease Steps (APDDS) Scale score of 1 or ≥6 (equivalent to an Expanded Disability Status Scale score of 1 or ≥6); (ii)	**Study design**<br>Single-centre wait-list randomised controlled study<br><br>**Intervention**<br>N=15<br>Mii-VitaliSe (plus usual care): A home-based, physiotherapist-supported Nintendo Wii intervention. The intervention consisted of two supervised Nintendo Wii familiarisation sessions in the hospital followed by home use (Wii Sports, Sports Resort and Fit Plus software) with physiotherapist support and personalised resources. Researcher also provided motivational interviewing to participants.<br>Participants were provided with commercially available software (Wii Fit Plus, Wii Sports and Wii Sports Resort) along with the Wii balance board (and non-slip cover), two Wii remote controls, two Nunchuk controls.<br>Participants were also provided with a 'Play Log' that tracked activity/progress.<br>**Control/comparator**<br>N=15<br>Wait-list control (WLC): Participants waited 6-months before receiving the	**Physical activity**<br>*Godin Leisure-Time Exercise Questionnaire (GLTEQ):*<br>Self-report measure of usual physical activity.<br>Frequency and intensity of exercise during free time in a typical week is measured.<br>Weekly frequencies are multiplied by metabolic equivalents to calculate total leisure activity.<br><br>Second question asks about the frequency of engaging in any	**Physical activity outcomes**<br>*Godin Leisure-Time Exercise Questionnaire (GLTEQ):*<br><br>At 6-months (mean, SD)<br><br>Mii group: 22.46, (16.39)<br>WLC: 11.20, (9.77)<br>Mean diff: 8.32 (−2.01 to 18.65)<br>Effect size: 0.70<br><br>At 12 months<br>Mii group: 28, 24.61<br>WLC: 18.17, 17.57<br>No effect size reported	**Cost**<br>Mean cost of delivering Mii-vitaliSe was £684 per person.<br>This includes cost of:<br>- delivery from Physiotherapist (£384 per participant)<br>- cost of Wii equipment (£300 per unit)

Authors<br>Country<br>Years data collected	Setting<br>Sample characteristics (sample size, demographics)	Recruitment method<br>Eligibility criteria	Study design<br>Virtual reality intervention description<br>Control/comparators description<br>Retention at follow-up/end of treatment	Measures of health behaviours	Health behaviours outcomes	Secondary Outcomes<br><br>Use of support received<br>Satisfaction/acceptability of support received<br>Costs
	Married/cohabiting	a relapse within the past 3 months that required treatment with corticosteroids and/or a hospital admission; (iii) already participating in exercise or rehabilitation research; (iv) a medical condition placing an individual at risk from exercise participation; (vi) owns a Wii and is currently using it on a weekly basis or more; (vii) unwilling or unable to comply with the protocol (eg, long vacation planned).	intervention.<br><br>**Retention**<br>97% (29/30) retained at 6-months<br>93% (28/30) retained at 12-months.	regular activity long enough to work up a sweat with three response options provided (often, sometimes, never/rarely).		
Ulas and Semin [40]<br>Turkey<br>NA	Volunteer students from the International Izmir University, Turkey (n=80)<br><br>**Sex**<br>55% (44/80) female<br>**Age (mean, SD)**<br>Control: 19.09 ± 0.81<br>VRE: 19 ± 0.72<br>TE: 19.12 ± 0.19<br>**BMI (kg/m2)**	Mail sent to all students of the vocational school of health sciences.<br><br>**Eligibility criteria**<br>between 18 and 25 years old, having no chronic diseases	**Study design**<br>RCT<br><br>**Intervention**<br>N=30<br>Virtual reality exercise group (VRE): Virtual reality headset and activity monitors were equipped to participants. Moderate exercise sessions were done 30 minutes/session, 3 times a week, for 8 weeks.<br>Participants determined the intensity of the exercises.<br>Participants watched and copied the exercises through the headset. Exercises	**Physical activity**<br>Activity monitor: step counts, active energy consumption (kcal) and physical activity level (Mets) (PAL).<br><br>Pulse oximeter: oxygen saturation.	**Physical activity outcomes**<br>*Activity monitor (energy consumption, step count and PAL)*<br>**Mean scores during exercise**<br>PAL (mean, SD)<br>VRE: 64.66, 3.71 Mets<br>TE: 68.34, 5.90 Mets<br>S (P < .003)<br><br>Active energy consumption (mean, SD)	NA

Authors Country Years data collected	*Setting Sample characteristics (sample size, demographics)*	*Recruitment method Eligibility criteria*	*Study design Virtual reality intervention description Control/comparators description Retention at follow-up/end of treatment*	*Measures of health behaviours*	*Health behaviours outcomes*	*Secondary Outcomes*<br><br>*Use of support received Satisfaction/acceptability of support received Costs*
	**(mean, SD)**<br>Control: 20.85 ± 1.74<br>VRE: 22.60 ± 3.44<br>TE: 22.20 ± 2.51		performed were, step aerobics, sit to stand, sit to reach. Each exercise was done twice for 5 minutes.<br>**Control/comparator**<br>N=30<br>Traditional exercise group (TE): Participants performed the same exercises as the VRE group, and exercised in front of a mirror, with the program displayed on a monitor.<br><br>N=20<br>Control group: avoid exercise, except daily routine tasks for 8 weeks.<br><br>**Retention**<br>Retention rates and dropouts not reported		VRE: 267.48, 71.28 Kcal<br>TE: 360.72, 53.28 Kcal<br>S (p < 0.000)<br><br>Step counts (mean, SD)<br>VRE: 2545.77, 678.32<br>TE: 3398.59, 529.19<br>S (p < 0.000)<br><br>*Pulse oximeter measures*<br>**Post-exercise means**<br>Pulse (mean, SD)<br>VRE: 99.91, 15.76<br>TE: 115.17, 18.16<br>S (p < .05)<br>Oxygen saturation (mean, SD)<br>VRE: 99.79, 0.47<br>TE: 99.81,0.35<br>NS<br><br>*Post intervention 6-month follow-up*<br>Proportion doing fitness, training 4 times/week for an average of 1.75 hrs/week<br>CG: 20% (4/20)<br>VRE: 20% (6/30)<br>TE: 16.7% (5/30)<br>(sig not reported)	

Authors<br>***Country***<br>***Years data collected***	***Setting***<br>***Sample characteristics (sample size, demographics)***	***Recruitment method***<br>***Eligibility criteria***	***Study design***<br>***Virtual reality intervention description***<br>***Control/comparators description***<br>***Retention at follow-up/end of treatment***	***Measures of health behaviours***	***Health behaviours outcomes***	***Secondary Outcomes***<br><br>***Use of support received***<br>***Satisfaction/acceptability of support received***<br>***Costs***
Villafaina et al. [41]<br>Spain<br>2017	Participants were from Cáceres, Spain. (N=55)<br><br>Analysis only performed on participants remaining at 48-week follow-up (n=37)<br>**Sex**<br>100% (37/37) female<br>**Age (mean, SD)**<br>Exergame: 54.27, 9.29<br>Control: 53.44, 9.47<br>**BMI (mean, SD)**<br>Exergame: 27.11, 2.90<br>Control: 28.19, 3.88	Recruited from a local Fibromyalgia (FM) association in Cáceres, Spain.<br><br>**Eligibility criteria**<br>Female and aged between 30 and 75 years; able to communicate with research staff; had given their informed consent; and diagnosed with FM by a rheumatologist according to 2010 American College of Rheumatology criteria.<br><br>Excluded if they (a) had changed their usual care therapies during the 24 weeks of the treatment, (b) had contraindications for physical-exercise programs, or (c) were pregnant.	**Study design**<br>Single-blinded RCT<br><br>**Intervention**<br>N=28<br>Exergame: exergame (VirtualEx-FM) group conducted a 24-week exercise intervention with 2 sessions per week, each of 1 h duration.<br>A typical session involved:<br>• A warm-up where participants were guided by a video made by a kinesiologist;<br>• aerobic exercises based on dance steps shown by a dance teacher;<br>• postural control and coordination games where participants had to reach for an apple that came and went in different locations around them (the kinesiologist could manually control the body part that participants had to use to reach the apple); and<br>• walking training where participants had to follow a virtual trail of footprints. The type and amplitude of steps were controlled.<br><br>**Control/comparator**<br>N=27<br>The control group continued with their usual daily life. This included remaining on medications.	**Physical activity**<br>*The International Physical Activity Questionnaire (IPAQ)*: monitor physical activity and inactivity. Used to calculate total Metabolic Equivalents (METs) per week and sitting time (minutes per day).	**Physical activity outcomes**<br>*IPAQ (METS per week) (mean, SD)*<br>24-weeks<br>Exergame: 2990.17, 3090.58<br>Control: 3406.94, 6315.86<br>NS<br><br>48-weeks<br>Exergame: 3194.14, 3356.59<br>Control: 3063.29, 2942.56<br>NS	NA

Authors<br>*Country*<br>*Years data collected*	*Setting*<br>*Sample characteristics (sample size, demographics)*	*Recruitment method*<br>*Eligibility criteria*	*Study design*<br>*Virtual reality intervention description*<br>*Control/comparators description*<br>*Retention at follow-up/end of treatment*	*Measures of health behaviours*	*Health behaviours outcomes*	*Secondary Outcomes*<br><br>*Use of support received*<br>*Satisfaction/acceptability of support received*<br>*Costs*
			**Retention**<br>91% (50/55) retained at end of 24-week intervention.<br>67% (37/55) retained at 48-week follow-up.			
Wilzeck et al. [42]<br>Switzerland<br>2014	Patients or outpatients at Cantonal Hospital of Baden, Switzerland (N=108)<br><br>N=86 completed trial and analysed<br>**Sex**<br>42% (36/86) female<br>**Age (mean, SD)**<br>Video group: 60.6, 16.3<br>Control group: 60.4, 15.1<br>**BMI (mean, SD)**<br>Video group: 27.0, 4.2<br>Control group: 27.1, 5.1<br>**Smoking status**<br>30% (26/86) current smokers<br>**Outpatients**<br>Video group:	Participants were recruited from a teaching hospital and outpatient hospital.<br>Every consecutive patient from June to September 2014 who did not meet the exclusion criteria and gave their informed consent was included in the trial.<br><br>**Eligibility criteria**<br>Participants were adults, either hospitalised or outpatients, at a Swiss teaching hospital with an indication for exercise treadmill testing.<br>Exclusion criteria were any criteria presenting a contraindication for exercise testing and the physical inability to perform an exercise treadmill test.	**Study design**<br>Randomised open-label controlled trial<br><br>The protocol consisted of one minute of walking at 1.2km/h without a gradient to allow the patients to get used to walking on a treadmill. Afterwards, the speed and gradient were increased gradually every three minutes for 7 iterations.<br>Exercise termination was determined by:<br>- ECG signs of myocardial ischemia<br>- a decrease in systolic blood pressure of at least 10 mm Hg,<br>- systolic blood pressure higher than 250/120 mm Hg, higher graded arrhythmias<br>- and the patient's request to stop.<br>**Intervention**<br>N=54<br>Video group: Exercise treadmill test while watching a virtual walking group on a screen in front of the treadmill. The video showed five amateur walkers	**Physical activity**<br>*Age-predicted METs*: Exercise capacity.<br>The achieved METs were calculated by the treadmill system.<br><br>*Exercise duration:* how many minutes before the participant interrupted the test due to exhaustion.	**Physical activity outcomes**<br>*Percentage of age-predicted peak METs achieved by participants (%, SD)*<br>Video group: 149, 32<br>Control group: 135, 29<br>S ($p < 0.041$)<br><br>*Exercise duration (average test duration in mins).*<br>Video group: 11:12, 2:54<br>Control group: 8:54, 2:39<br>S ($p < 0.001$)	NA

Authors<br>*Country*<br>*Years data collected*	*Setting*<br>*Sample characteristics (sample size, demographics)*	*Recruitment method*<br>*Eligibility criteria*	*Study design*<br>*Virtual reality intervention description*<br>*Control/comparators description*<br>*Retention at follow-up/end of treatment*	*Measures of health behaviours*	*Health behaviours outcomes*	*Secondary Outcomes*<br><br>*Use of support received*<br>*Satisfaction/acceptability of support received*<br>*Costs*
	88.4% (38/43)<br>Control group: 83.7% (36/43)		walking on a path along woods and fields, giving the patient the perspective of a person walking with the group. The walking speed of the people in the video correlated with the walking speed of the treadmill.<br><br>**Control/comparator**<br>N=54<br>Control group: watched a static image of lavender flowers during exercise.<br><br>**Retention**<br>80% (86/108) retained at end of trial (1 day).			

<u>Physical Activity, Obesity/Weight</u>

Adamo et al [49]<br>Canada<br>2007-2009	Endocrinology clinic at the Children's Hospital of Eastern Ontario (Ottowa) n=30<br><br>Baseline data excluded dropouts (n=26)<br>**Sex**<br>60% (14/26) Male<br>**Age (mean, SD)**<br>Music group =	150 families were screened through the Endocrinology clinic at the Children's Hospital of Eastern Ontario (CHEO; Ottawa, Ont.)<br><br>**Eligibility criteria**<br>Youth between aged 12–17 years, body mass index (BMI) above the 95th percentile or a BMI > 85th percentile for age and	**Study Design**<br>Randomised controlled clinical trial (RCT)<br><br>**Intervention**<br>N=13<br>Interactive video game cycling (VG): two 60-min sessions per week for 10 weeks. GameBike interactive video gaming system interfaced with a Sony Playstation 2 and a 42" Television monitor. Using a handlebar mounted controller Participants played a selection	**Physical Activity**<br>*Heart rate*: polar heart rate monitor<br>*Training intensity*: average number of minutes per session spent at vigorous intensity (80-	**Physical activity outcomes**<br>*Peak heart rate (beats-min)*<br>Significant within-group improvements in peak HR ($p = 0.004$) (i.e., reduction in peak HR at peak workload), peak workload ($p = 0.038$), and time to exhaustion	**Attendance and adherence**<br>Percentage of the 20 sessions attended (mean percent, SD)<br>MG: 92.3%, 3.9%<br>VG: 86.1%, 5.8%<br>S ($p < 0.05$).

Authors<br>***Country***<br>***Years data collected***	***Setting***<br>***Sample characteristics (sample size, demographics)***	***Recruitment method***<br>***Eligibility criteria***	***Study design***<br>***Virtual reality intervention description***<br>***Control/comparators description***<br>***Retention at follow-up/end of treatment***	***Measures of health behaviours***	***Health behaviours outcomes***	***Secondary Outcomes***<br><br>***Use of support received***<br>***Satisfaction/acceptability of support received***<br>***Costs***
	15.1, 1.8<br>Video Group=13.9, 1.4<br>**Weight (kg)**<br>88% (23/26) Obese<br>22% (3/26) Overweight	gender with elevated fasting glucose or 2-h Oral Glucose Tolerance Test (OGTT), elevated fasting triglycerides, insulin, high-density lipoprotein cholesterol (HDL-C), low-density lipoprotein cholesterol (LDL-C), or total cholesterol/HDL-C > 90th percentile, blood pressure above the 90th percentile, or first degree relative with type 2 diabetes or CVD. Do not have a medical condition that altered intestinal absorption or otherwise influence response to activity intervention or make vigorous exercise dangerous, or any other illness assessed by their physician making participation inadvisable, were considered eligible to participate.<br>Were not taking performance enhancing medication, medication or supplement that could affect body composition,	of interactive race-based games while cycling. The faster participants pedalled the faster they moved in the virtual world on screen.<br><br>**Control/Comparator**<br>N=13<br>Music group (MG): Stationary bike music-comparison group: exercised twice weekly for 10 weeks on the GameBike with the game console turned off.<br>Participants could listen to music while exercising.<br>**Retention**<br>87% (26/30) remained at 10-weeks	100% of predicted maximum HR)<br><br>*Distance travelled (km):* tracked by GameBike<br><br>*Duration pedalled (min)*: average minutes per session spent pedalling<br><br>**Obesity**<br>Body weight and fat: body mass index (BMI)	(p = 0.038).<br><br>*Training intensity*<br>Average minutes spent in 80-100% peak HR – vigorous intensity (mean, SD)<br>VG: 13.7, 12.8<br>MG: 24.9, 20.0<br>S (p<0.05)<br><br>Average minutes spent in 60-79% peak HR – moderate intensity (mean, SD)<br>VG: 37.4, 12.3<br>MG: 29.6, 17.5<br>NS<br><br>*Average Distance travelled (km) (mean, SD)*<br>VG: 10.3, 2.2<br>MG: 12.5, 2.8<br>S (p<0.03)<br><br>*Duration pedalled (mins) (mean, SD)*<br>VG: 54.3, 16.9<br>MG: 56.3, 4.1<br>NS<br><br>**Obesity outcomes**	

Authors Country Years data collected	*Setting Sample characteristics (sample size, demographics)*	*Recruitment method Eligibility criteria*	*Study design Virtual reality intervention description Control/comparators description Retention at follow-up/end of treatment*	*Measures of health behaviours*	*Health behaviours outcomes*	*Secondary Outcomes*<br><br>*Use of support received Satisfaction/acceptability of support received Costs*
		lipids, or glucose metabolism, weight increase of 10% or decrease of 5% or more in the last 2 months, participated in a regular exercise program or sport in the last 4 months, planning on starting an exercise program outside the study, presented with an eating disorder, depression, or abusing alcohol or drugs.			BMI (mean, SD)<br>VG: 35.5, 9.7<br>MG: 39.4, 8.9<br>NS<br>No significant group or group time effects on body weight, body mass index, fat mass, fat-free mass, or waist circumference.	

Obesity/Weight

Behm-Morawitz, Lewallen, Choi [43]<br>USA<br>NA	Missouri University, in the city of Columbia, Missouri (n=92).<br><br>Analysed (n=90)<br>**Sex**<br>98% (90/92) female<br>**Age (mean, SD)**<br>25, 9.92<br>**Ethnicity**<br>76% (70/92) White	92 participants recruited using mass email at Missouri University, and flyers in the City of Missouri.<br><br>**Eligibility criteria**<br>Overweight adults seeking to lose weight healthfully.	**Study design**<br>Randomized controlled trial (RCT)<br><br>**Intervention**<br>N=NA<br>Second Life (SL): 3D social virtual world (avatar virtual interaction). Using a home computer, participants used a virtual avatar in the program Second Life, to visit a list of locations within a virtual world that promoted physical activities.<br>Participants signed up to use the virtual world in groups of 3-4 but spent 75% of	**Obesity**<br>Average weight loss (pounds)	**Obesity outcomes**<br>Mean weight loss (pounds) at end of treatment (4-weeks):<br>SL: 1.75<br>Control: 0.91<br>S (p = 0.04)	NA

Authors Country Years data collected	*Setting Sample characteristics (sample size, demographics)*	*Recruitment method Eligibility criteria*	*Study design Virtual reality intervention description Control/comparators description Retention at follow-up/end of treatment*	*Measures of health behaviours*	*Health behaviours outcomes*	*Secondary Outcomes*<br><br>*Use of support received Satisfaction/acceptability of support received Costs*
	13% (12/92) African American/black<br>10% (9/92) Asian<br>4% (4/92) Hispanic/ Latino<br>1% (1/92) Other		their time in SL engaging in independent activities.<br>Participants played a minimum of twice a week (total 2hrs/week) for 4 weeks. Over 90% of SL participants reported spending an average of 2hrs/week in the virtual world, with little variance.<br><br>**Control/ comparator**<br>N=NA<br>Control: 2D social networking site (no avatar virtual interaction control condition): a virtual health social networking site created using Yooco.com was explored twice a week on average. Participants could also respond to post on health and nutrition.<br><br>No intervention (no virtual interaction control condition). Both control groups were collapsed into one for analysis.<br><br>**Retention**<br>39% (36/92) of participants completed post-test measures (week 4).			
Cesa et al. [44]<br>Italy<br>NA	Eating Disorder Unit of the Istituto Auxologico Italiano, Verbania, Italy (n=90)<br><br>Baseline	Consecutive patients seeking treatment at the Eating Disorder Unit of the Istituto Auxologico Italiano, Verbania, Italy, were seen for screening interviews for admission	**Study design**<br>Clinical RCT<br><br>All participants received the <u>6-week</u> inpatient treatment program. This was all the control group received.<br>**Intervention**	**Obesity/weight**<br>Weight: kg, BMI	**Obesity/weight outcomes**<br>*Weight significantly decreased in all the three conditions at end of treatment*<br>ECT: -6.17 kg, CI -7 to -	NA

Authors Country Years data collected	***Setting Sample characteristics (sample size, demographics)***	***Recruitment method Eligibility criteria***	***Study design Virtual reality intervention description Control/comparators description Retention at follow-up/end of treatment***	***Measures of health behaviours***	***Health behaviours outcomes***	***Secondary Outcomes<br><br>Use of support received Satisfaction/acceptability of support received Costs***
	demographics reported (N=66)<br>**Sex**<br>100% (66/66) female<br>**Age (mean, SD)**<br>31.79, 7.9 years<br>**Weight (mean, SD)**<br>106.6, 17.7 kg<br>**Height (mean, SD)**<br>162, 7 cm<br>**BMI (mean, SD)**<br>40.5, 5.2<br><br>**Education (high school)**<br>ECT: 51.9% (n=14/27)<br>CBT: 60% (n=12/20)<br>IP: 73.7% (n=14/19)	to the study.<br><br>**Eligibility criteria**<br>(1) women aged 18-50 years, (2) who met DSM-IV-TR criteria for Binge-eating disorder (BED) for at least 6 months prior to the beginning of the study, (3) no other concurrent severe psychiatric disturbance (psychosis, depression with suicidal risk, alcohol or drug abuse), (4) no concurrent involvement in other treatment for BED, including pharmacotherapy, (5) no concurrent medical condition not related to the disorder, and (6) written and informed consent to participate.	N=31<br>*VR-Enhanced Cognitive Behaviour Therapy*:<br>(*CBT*): Received 15 additional sessions over 5 weeks. After the first inpatients week, participants entered five weekly group sessions similar to the CBT ones (focused on concerns about body weight and shape and problematic eating) and 10 biweekly VR sessions.<br>NeuroVR software was used for VR, which includes 14 virtual environments used by the therapist during a 60-minute session with the patient. The environments present critical situations related to the maintaining/relapse mechanisms (home, supermarket, pub, restaurant, swimming pool, beach, gymnasium) and two body-image comparison areas. Through the VR experience, patients practice both eating/emotional/ relational management and general decision-making and problem-solving skills.<br>The first session was used to assess any stimuli that could elicit abnormal eating behavior. Specifically, the attention was focused on a patient's concerns about food, eating, shape, and weight. The next 14 sessions were used to assess and modify:<br>- Expectations and Emotions Related to Food and Weight		5.3, P< .001;<br>CBT: -7.1 kg, CI -7.9 to -6.2, P<. .001;<br>IP: -6.6 kg, CI -8.1 to -5.2, P<. 001)<br>without any significant differences between them.<br><br>**BMI**<br>*End of treatment to 1-year follow-up (median, SD)*<br>ECT: 36.2, 5<br>CBT: 39.1, 3.6<br>IP: 41.5, 6<br>S (p<.015)<br>Only VR was effective at improving weight loss at 1-year follow-up.<br><br>Note: increase BMI in control<br>IP: 39.3 –40.9<br>S(P<.001)<br><br>*Baseline to 1-year follow-up (mean diff)*<br>ECT: 39.2 – 36.6 (-2.6)<br>CBT: 41.1 – 39 (-2.1)<br>IP: 41.8 – 40.9 (0.9)<br>S (p=0.052)	

Authors<br>*Country*<br>*Years data collected*	*Setting*<br>*Sample characteristics (sample size, demographics)*	*Recruitment method*<br>*Eligibility criteria*	*Study design*<br>*Virtual reality intervention description*<br>*Control/comparators description*<br>*Retention at follow-up/end of treatment*	*Measures of health behaviours*	*Health behaviours outcomes*	*Secondary Outcomes*<br><br>*Use of support received*<br>*Satisfaction/acceptability of support received*<br>*Costs*
			- Strategies Used to Cope With Difficult Interpersonal and Potential Maintenance Situations<br>- Body Experience of the Subject<br><br>VR body-image comparison used as guided imagery.<br><br>**Control/comparator**<br>N=30<br>*CBT:* Patients were taught to self-monitor their food intake and eating patterns thoughts, as well as the circumstances and environment surrounding eating (e.g., whether eating alone or with others, speed of eating, and place of eating). Patients were also taught to identify problems in eating, mood, and thinking patterns and to develop alternative patterns gradually. Patients entered five weekly group sessions, and 10 biweekly individual sessions.<br>The first 8 individual sessions were structured according to Stage 1 of the CBT manual for binge eating. They focused on an overview of the goals of the treatment program, the use of self-monitoring records to identify high-risk situations that might trigger binge eating, support in normalizing eating patterns, and the identification of		Post-hoc<br>ECT vs IP: P=.027<br><br>*% Participants weight maintenance or further loss at 1-year follow-up:*<br>ECT: 44.4%<br>CBT: 40%<br>IP: 10.5%<br>S (see below for comparisons)<br><br>ECT vs IP<br>(OR 6.8, 95% CI 1.3-35.4, P=.014).<br><br>CBT vs IP<br>(OR 5.7, 95% CI 1.09-31.5, P=.035).	

Authors Country Years data collected	*Setting Sample characteristics (sample size, demographics)*	*Recruitment method Eligibility criteria*	*Study design Virtual reality intervention description Control/comparators description Retention at follow-up/end of treatment*	*Measures of health behaviours*	*Health behaviours outcomes*	*Secondary Outcomes<br><br>Use of support received Satisfaction/acceptability of support received Costs*
			behavioural strategies for coping with high-risk situations for binge eating. The final 2 individual sessions focused on the maintenance of improvement and on relapse prevention. The group sessions were structured according to Stage 2 of the CBT manual for binge eating. They focused on problem-solving strategies and cognitive interventions targeting concerns about body weight and shape and problematic eating.<br><br>N=29<br>*Inpatient Program (IP):*<br>Hospital-based living for 6 weeks. Inpatients received medical, nutritional, physical, and psychological care. In particular, they maintained a low-calorie diet (tailored to patients' needs), entered weekly nutritional groups held by dieticians, received psychological support both in individual and group settings, and undertook physical training (30 minutes of walking two times a week as a minimum).<br><br>**Retention**<br>73% (66/90) patients completed treatment, 24 declined to participate. 49% (44/90) patients that were allocated to groups completed 1-year follow-up.			

Authors<br>***Country***<br>***Years data collected***	***Setting***<br>***Sample characteristics (sample size, demographics)***	***Recruitment method***<br>***Eligibility criteria***	***Study design***<br>***Virtual reality intervention description***<br>***Control/comparators description***<br>***Retention at follow-up/end of treatment***	***Measures of health behaviours***	***Health behaviours outcomes***	***Secondary Outcomes***<br><br>***Use of support received***<br>***Satisfaction/acceptability of support received***<br>***Costs***
Ferraz et al. [45]<br>Brazil<br>2015-2017	State Reference Health Care Centre for Elderly (SRHCCE), a public reference outpatient clinic for the elderly in Salvador, Bahia, Brazil (n=72)<br><br>Baseline and analysis reported (n=62)<br>**Sex**<br>60% (37/62) male<br>**Age years (mean, SD)**<br>69, 5<br>**Weight (kg) (mean, SD)**<br>67.05, 12.46<br><br>**Height (m) (mean, SD)**<br>1.61, 0.09<br><br>**Duration of illness (years) (mean, SD)**<br>6, 4<br><br>**BMI (kg/m²) (mean, SD)**	Participants were recruited from a public reference outpatient clinic for the elderly.<br><br>**Eligibility criteria**<br>Elderly patients (≥60 years of age) with idiopathic Parkinson's Disease (PD) according to the London Brain Bank criteria participated.<br><br>The inclusion criteria were regular use of medication for PD and modified Hoehn and Yahr stages 2, 2.5, or 3 (the interventions were developed for patients with bilateral disease involvement without severe disability) without walking devices. The exclusion criteria were visual or hearing impairment; parkinsonian syndromes other than PD; bone, joint, or muscle diseases that limit the practice of physical activity; chronic uncontrolled diseases (hypertension, diabetes	**Study design**<br>Clinical RCT<br><br>**Intervention**<br>N=22<br>Exergames:<br>Trained using Xbox 360 video game with Kinect. The Kinect Adventures games were used. These exergames use full-body motion to allow the player to engage in a variety of mini-games, all of which feature jump-in, jump-out multiplayer play. Each mini-game lasts about 3 minutes. To complete 30 minutes of training, the same 1 or 2 mini-games were repeated in different levels of intensity in each session.<br><br>**Control/comparator**<br>N=25<br>*Functional training*:<br>Functional training consisted of 10 activities lasting 3 minutes each one:<br>- Gait with obstacles<br>- Going up and down stairs and ramp<br>- Sitting and standing exercises<br>- Side gears<br>- Balance exercise in proprioceptive platform<br>- Activities with balls (e.g., kicking, lifting)<br>- Step exercises	**Obesity/weight**<br>*BMI (kg/m²)*	**Obesity/weight outcomes**<br>No groups decreased body mass index significantly<br>BMI (mean, SD)<br>Function training: 26.5, 4.1<br>Bicycle exercise: 24.1, 3.8<br>Xbox kinect: 26.3, 5.7<br>NS	NA

Authors ***Country*** ***Years data collected***	***Setting*** ***Sample characteristics (sample size, demographics)***	***Recruitment method*** ***Eligibility criteria***	***Study design*** ***Virtual reality intervention description*** ***Control/comparators description*** ***Retention at follow-up/end of treatment***	***Measures of health behaviours***	***Health behaviours outcomes***	***Secondary Outcomes*** <br>***Use of support received*** ***Satisfaction/acceptability of support received*** ***Costs***
	Function training: 26.62 (4.17)36 Bicycle exercise: 24.36 (3.97)36 Xbox kinect: 26.61 (5.83)36 <br>**Education (years) (mean, IR)** Function training: 8.00 (4.75-11.00) Bicycle exercise: 9.50 (5.00-11.00) Xbox kinect: 8.00 (4.00-11.00)	mellitus, chronic pain); unstable cardiovascular disease (acute heart failure, recent myocardial infarction, unstable angina, and arrhythmias uncontrolled); current alcohol and other toxic substance use; contraindications for performing physical exercise according to the American College of Sports Medicine; practicing any physical exercise program in the past 6 months, or participating in regular resistance training in the previous 12 months.	- Foot tip exercises - Graded reaching activities - Gait training <br>*Bicycle exercise*: N=25 Performed aerobic training on a stationary bicycle. In the first week, training was titrated to 50% of maximum heart rate, increasing progressively to 75% in the eighth week. <br>**Retention** 86% (62/72) retention at the end of the 8-week intervention.			
Manzoni et al. [46] Italy NA	The obesity unit of the Istituto Auxologico Italiano, Verbania, Italy, (n=163), <br>**Sex** 100% (163/163) female <br>**Age (mean, SD)** 35.63, 8.04	300 consecutive obese inpatients admitted to the obesity unit of the Istituto Auxologico Italiano, Verbania, Italy, for the treatment of obesity and related comorbidities. <br>**Eligibility criteria** (a) a BMI ≥40; (b) 18–50 years of age; (c) no other concurrent severe eating (bulimia, binge eating, or	**Study design** Randomised Controlled Clinical Trial <br>**Intervention** N=57 VR-enhanced *Cognitive-behavioural therapy (CBT*): Received 15 additional sessions over 5 weeks. After the first inpatients week, participants entered five weekly group sessions similar to the CBT ones (focused on concerns about body weight and shape and problematic eating) and	**Obesity** *Weight loss*: kg	**Obesity outcomes** Significant weight loss for the whole sample (effect size 0.72). <br>*Weight (kg) at baseline, post-treatment, 12-month follow-up (mean, SD)* **Baseline**: Control: 110, 15.2 CBT: 108, 12.1 VR: 112.1, 15.6	NA

Authors<br>*Country*<br>*Years data collected*	*Setting*<br>*Sample characteristics (sample size, demographics)*	*Recruitment method*<br>*Eligibility criteria*	*Study design*<br>*Virtual reality intervention description*<br>*Control/comparators description*<br>*Retention at follow-up/end of treatment*	*Measures of health behaviours*	*Health behaviours outcomes*	*Secondary Outcomes*<br><br>*Use of support received*<br>*Satisfaction/acceptability of support received*<br>*Costs*
	**Morbid obese (BMI >40) =** 100%<br>**Weight (mean, SD)**<br>110.33, 12.27kg<br>**Height (mean, SD)**<br>1.61, 0.03m<br>**BMI (mean, SD)**<br>42.24, 6.01<br>**Schooling**<br>Graduated from upper secondary school 51%<br>**Employment**<br>68.2% employed	eating disorder not otherwise specified) or psychiatric disturbances (psychosis, depression with suicidal risk, or alcohol or drug abuse); (d) no concurrent involvement in other treatment, including medication; (e) no concurrent medical condition not related to the disorder; and (f) written and informed consent to participate.	10 biweekly VR sessions.<br>NeuroVR software was used for VR, which includes 14 virtual environments used by the therapist during a 60-minute session with the patient. The environments present critical situations related to the maintaining/relapse mechanisms (home, supermarket, pub, restaurant, swimming pool, beach, gymnasium) and two body-image comparison areas. Through the VR experience, patients practice both eating/emotional/ relational management and general decision-making and problem-solving skills.<br><br>*Session 1*: assess stimuli elicit abnormal eating behaviour. Concerns about food, eating, shape, weight. Therapist uses VR to help participants imagine themselves as a lower weight.<br><br>*Session 2-10*: expectations and emotions related to food and weight. Recognise why they eat, plus CBT techniques to avoid or cope with emotional/behavioural triggers.<br><br>VR body-image comparison used as guided imagery. Patient to give detailed descriptions of the virtual experience and of the feelings associated with it. Furthermore, the patient is taught how		NS<br><br>**Post**:<br>Control: 103.2, 14.5<br>CBT: 100.5, 11.3<br>VR: 105, 14.3<br>NS<br><br>**Follow-up:**<br>Control: 114.7, 19.3<br>CBT: 105.8, 17.1<br>VR: 105.4, 16.2<br>Not performed due to dropout<br><br>*Average % of starting body weight lost from baseline to post-treatment.*<br>Control: 6.2%<br>CBT: 7.4%<br>VR: 6.25%<br>NS<br><br>*Effect size (Hedges g): start to 12-month follow-up*<br>Control: 0.277<br>CBT: -0.151<br>VR: -0.472<br><br>*Weight decrease from baseline to Follow-up*	

Authors Country Years data collected	*Setting Sample characteristics (sample size, demographics)*	*Recruitment method Eligibility criteria*	*Study design Virtual reality intervention description Control/comparators description Retention at follow-up/end of treatment*	*Measures of health behaviours*	*Health behaviours outcomes*	*Secondary Outcomes*<br><br>*Use of support received Satisfaction/acceptability of support received Costs*
			to cope with them using different techniques.<br><br>**Control/comparator**<br>N=54<br>*CBT:* Patients were taught to self-monitor their food intake and eating patterns thoughts, as well as the circumstances and environment surrounding eating (e.g., whether eating alone or with others, speed of eating, and place of eating). Patients were also taught to identify problems in eating, mood, and thinking patterns and to develop alternative patterns gradually. In particular, after the first week, the patients entered five weekly group sessions aimed at addressing weight and primary goals, and 10 biweekly individual sessions aimed at establishing and maintaining weight loss, addressing barriers to weight loss, increasing activity, addressing body image concerns, and supporting weight maintenance<br><br>N=52<br>*Standard behavioural program (SBP) (control):* It consists of hospital-based living for <u>6 weeks</u>. Inpatients receive medical, nutritional, physical, and psychological care the goal of which is to provide practical guidelines (e.g.,		Within group mean scores<br>VR: S<br>CBT: NS<br>SBP: NS<br><br>*Weight regain from Post to follow-up*<br>VR: NS<br>CBT: NS<br>SBP: S<br><br>*Weight increase from Post to Follow-up*<br>Post-hoc between group<br>VR vs SBP = S<br>CBT vs SBP = S<br>VR vs CBT = NS<br><br>*Maintaining or improving weight loss at 1-year* (Probabilities – odds ratios)<br>VR: [22/46, 48%]<br>SBP: [3/26, 11.5%]<br>OR 7.03 [95% CI 1.85–26.70])<br><br>VR: [22/46, 48%]<br>CBT: [11/38, 29%]<br>OR 2.25 [95% CI 0.91–5.58]).	

Authors Country Years data collected	*Setting Sample characteristics (sample size, demographics)*	*Recruitment method Eligibility criteria*	*Study design Virtual reality intervention description Control/comparators description Retention at follow-up/end of treatment*	*Measures of health behaviours*	*Health behaviours outcomes*	*Secondary Outcomes*<br><br>*Use of support received Satisfaction/acceptability of support received Costs*
			stressing gradual weight loss with the caloric restriction achieved largely by reductions in fat intake), plus a low-calorie diet (1,200 kcal/day) and physical training (30 minutes of walking twice a week as a minimum).<br><br>**Retention**<br>69% (113/163) participants completed 1-year follow-up.<br>There was a significant between-group difference for retention with lower retention in the SBP group (p = 0.02).			
Manzoni et al. [47]<br>Italy<br>2007	San Giuseppe Hospital, Istituto Auxologico Italiano, Verbania, Italy. (n=60)<br><br>**Sex**<br>100**%** female<br>**Age (mean, SD)**<br>VR group: 42.8, 11.44<br>IM group: 48.55, 7.96<br>Control group: 39.65, 14.52<br><br>**BMI (mean, SD)**<br>VR group: 41.74, 3.94	Participants were recruited from an obese population admitted for weight reduction treatment at San Giuseppe Hospital, Istituto Auxologico Italiano, Verbania, Italy.<br><br>**Eligibility criteria**<br>Between ages 18 and 60 years; have received a primary diagnosis of obesity based on World Health Organization criteria; report the presence of recurrent episodes of emotional eating, as assessed	**Study design**<br>Three-arm exploratory randomized controlled trial<br><br>All women recruited for participation in the trial underwent a <u>5-week</u> hospital-based program for weight reduction and rehabilitation.<br>**Intervention**<br>N=20<br>*Virtual reality:*<br>Participants wore a head mounted display for immersion into the virtual environment. Common relaxation training protocol consisted of four sessions per week (12 sessions in total) and lasted 3 weeks. It included a combination of different relaxation techniques mainly based on Progressive	**Obesity/weight**<br>Weight (kg): measured using a balance scale for weight.	**Obesity/weight outcomes**<br>Weight (kg) significantly decreased within all three groups <u>without significant difference among them</u>.<br>VR group<br>Pre: 110, 6<br>Post: 109,5<br>Follow-up: 101, 5<br><br>Imagination group<br>Pre: 104, 9<br>Post: 98, 5<br>Follow-up: 99<br><br>Control group<br>Pre: 107, 7	NA

Authors Country Years data collected	*Setting Sample characteristics (sample size, demographics)*	*Recruitment method Eligibility criteria*	*Study design Virtual reality intervention description Control/comparators description Retention at follow-up/end of treatment*	*Measures of health behaviours*	*Health behaviours outcomes*	*Secondary Outcomes<br><br>Use of support received Satisfaction/acceptability of support received Costs*
	IM group: 41.82, 4.72<br>Control group: 43.21, 6.48<br><br>**Married (%)**<br>VR group: 50%<br>IM group: 70%<br>Control group: 45%<br><br>**Education between 8-13 years (%)**<br>VR group: 45%<br>IM group: 55%<br>Control group: 40%<br><br>**Employed (%)**<br>VR group: 55%<br>IM group: 50%<br>Control group: 55%	through the Emotional Overeating Questionnaire (EOQ); and have a body mass index ≥30. Patients affected by other psychiatric, psychological, or neurological disorders were excluded from the study	Muscular Relaxation and the Applied Relaxation technique. In both intervention conditions (imaginative condition and virtual reality), relaxation training was provided through recorded audio-narratives. In the virtual reality condition, narratives were presented together with a very relaxing virtual environment (named Green Valley) showing a mountain landscape around a calm lake. After being immersed in the Green Valley, participants were asked to walk around the lake, to observe the nature and, after few minutes, to virtually sit on a comfortable deck chair and relax.<br><br>**Control/comparator**<br>N=20<br>*Imaginative condition:*<br>Participants included in the IM group listened to the narrative with their eyes closed. In the imaginative condition, narratives suggest imagining a similar environment.<br><br>N=20<br>*Control condition:*<br>All patients receiving the standard treatment program were placed on a low-energy balanced diet (80% of basal energy consumption estimated according to the Harris-Benedict		Post: 96<br>Follow-up: 102	

Authors Country Years data collected	*Setting Sample characteristics (sample size, demographics)*	*Recruitment method Eligibility criteria*	*Study design Virtual reality intervention description Control/comparators description Retention at follow-up/end of treatment*	*Measures of health behaviours*	*Health behaviours outcomes*	*Secondary Outcomes<br><br>Use of support received Satisfaction/acceptability of support received Costs*
			equation) that consisted of 16% protein, 25% fat, and 59% carbohydrates, and participated in nutrition programs provided by a food and nutrition professional, psychological counseling provided by a clinical psychologist, and physical activity training provided by a physiotherapist. Nutrition training took place in group sessions (90 minutes each) twice a week and included information on obesity and its causes, evaluation of nutritional status, modalities for proper eating, regulation of body weight, biological and social stimuli affecting food intake, and strategies for engaging in regular physical exercise and for long-term weight management.<br><br>**Retention**<br>60% (36/60) retained at 3-month follow-up.			
Warburton et al. [48]<br>Canada<br>NA	Participants were from British Columbia, Canada.<br><br>N=14<br>**Sex**<br>100% (14/14) male<br>**Age (mean, SD)**<br>IVG: 23, 5	Participants were recruited using advertisements.<br><br>**Eligibility criteria**<br>Low-active young males (18–25 y), who were engaging in physical activity below Health Canada's recommended	**Study design**<br>RCT<br><br>Both groups received a recommended exercise regime:<br>Moderate intensity exercise (i.e., 60%–75% of heart rate reserve), 3 d/week for 30 min/d for 6 weeks.<br>Standardized warmup and cool-down period consisting of light stretching and	**Obesity/weight**<br>BMI: Body mass (kg) and standing height (cm) were measured according to standard procedures**.**	**Obesity/weight outcomes**<br>6-weeks<br>BMI (mean, SD)<br>IVG: 27, 6<br>SC: 27, 6<br>NS	**Adherence**<br>Mean % Attendance, SD<br>IVG: 78, 18<br>SC: 48, 29<br>S

Authors Country Years data collected	*Setting Sample characteristics (sample size, demographics)*	*Recruitment method Eligibility criteria*	*Study design Virtual reality intervention description Control/comparators description Retention at follow-up/end of treatment*	*Measures of health behaviours*	*Health behaviours outcomes*	*Secondary Outcomes<br><br>Use of support received Satisfaction/acceptability of support received Costs*
	SC: 22, 2<br>**Height (mean, SD)**<br>IVG: 177, 6<br>SC: 176, 10	threshold (4 times/week for at least 30 min at a moderate intensity) over the last 3 months.<br>Exclusion criteria included individuals who (i) were engaged in a regimented endurance training program within the previous 6 months, (ii) had participated in another research investigation within 30 d, (iii) exhibited abnormal blood pressure responses at rest (i.e., systolic blood pressure ‡140 mmHg and (or) diastolic blood pressure ‡90 mmHg) and (or) during exercise testing, and (iv) had known cardiopulmonary disease.	5 min of submaximal cycling at a low intensity (i.e., approximately 30% of heart rate reserve). Participants were permitted to engage in exercise during the weekdays (Monday– Friday) between 7 am and 7 pm. They also wore heart rate monitors on each training day and were provided individualized training heart rate (HR) zones. Individuals were able to self-monitor their HR.<br>**Intervention**<br>N=7<br>Interactive videogame (IVG): exercised on a GameBike1 interactive video gaming system that was linked to a Sony Playstation 2 and a television monitor. The GameBike1 system reads the participant's speed (measured by cycling cadence) and steering.<br>Playable video games included Smuggler's Run, ATV Offroad Fury, Gran Turismo 3, NASCAR Heat, and Need for Speed. They were instructed to exercise at a moderate intensity by manually adjusting the cycle ergometer resistance during each training session.<br>Participants could exercise at an intensity and duration that they desired.<br><br>**Control/comparator**<br>N=7<br>Stationary cycling (SC): exercised on a			

Authors<br>*Country*<br>*Years data collected*	*Setting*<br>*Sample characteristics (sample size, demographics)*	*Recruitment method*<br>*Eligibility criteria*	*Study design*<br>*Virtual reality intervention description*<br>*Control/comparators description*<br>*Retention at follow-up/end of treatment*	*Measures of health behaviours*	*Health behaviours outcomes*	*Secondary Outcomes*<br><br>*Use of support received*<br>*Satisfaction/acceptability of support received*<br>*Costs*
			standard bicycle at an intensity and duration that they desired. Identical to the intervention group, participants were provided a recommended exercise prescription<br><br>**Retention**<br>Retention rates not reported<br>Note: study mentions all participant data analysed, however not all participants attended each week, with attendance rates for the control group dropping each week.			

Obesity/Weight, Nutrition

Vieira et al [50]<br>Portugal<br>NA	Participants were from Centro Hospitalar do Porto (Porto Healthcare Center in Portugal). (n=46)<br><br>Baseline analysed (N=33)<br>**Sex**<br>100% (33/33) male<br>**Age (mean, SD)**<br>IG1: 55, 9.0	Subjects who had just completed the training phase of Cardiac Rehabilitation (CR) at the Cardiovascular Prevention and Rehabilitation Unit were invited to participate.<br><br>**Eligibility criteria**<br>Men and women, aged between 40 and 75 years, with coronary artery disease, diagnosed and stabilized, with no	**Study design**<br>Clinical RCT<br><br>Pamphlets with information on the risk factors for cardiovascular disease, which focused on eating habits, smoking and physical activity was delivered to the participants in the intervention groups. Program ran for 6-months with follow-up 3-months post-intervention.<br>**Intervention**<br>N=15<br>Intervention group 1 (IG1): home-based CR program, using a computer and Kinect (VR). Participant's movements	**Obesity/weight**<br>BMI<br><br>**Nutrition**<br>Semi-Quantitative Food Frequency Questionnaire, validated for the Portuguese population: self-report measures of frequency and	**Obesity/weight**<br>3-months<br>BMI (mean, SD)<br>N=30<br>IG1: 27.3, 3.6<br>IG2: 25.6, 2.8<br>CG: 27.7, 3.5<br>NS<br><br>6-months<br>BMI (mean, SD)<br>N=31<br>IG1: 27.4, 4.2<br>IG2: 25.9, 3.0<br>CG: 28.1, 3.5	**Adherence**<br>Attended 3 session a week<br>6-months (mean %)<br>IG1: 77%<br>IG2: 83%<br>NS

Authors Country Years data collected	*Setting Sample characteristics (sample size, demographics)*	*Recruitment method Eligibility criteria*	*Study design Virtual reality intervention description Control/comparators description Retention at follow-up/end of treatment*	*Measures of health behaviours*	*Health behaviours outcomes*	*Secondary Outcomes*<br><br>*Use of support received Satisfaction/acceptability of support received Costs*
	IG2: 59, 11.3<br>CG: 59, 5.8	unstable angina and complex ventricular arrhythmias with or without percutaneous coronary intervention and a final diagnosis of acute myocardial infarction or stable angina pectoris, that completed training phase of CR at the Cardiovascular Prevention and Rehabilitation Unit; and had access to a computer with Microsoft Windows 7 (minimum).<br>Exclusion criteria included heart surgery, non-completed stress test due to maximum fatigue, pregnancy or planning to get pregnant, cardiovascular high risk, pacemaker, severe neurological, musculoskeletal or pulmonary diseases, and, uncompensated metabolic disorders, reported dementia, cardiomyopathies and previous cardiorespiratory arrest non-associated with acute myocardial	are captured by sensors (Microsoft Kinect) and represented in game by an avatar. The system's virtual physical therapist performs the exercises and provides feedback on the participant's form.<br>10 min warmup<br>20-25 min strength exercise (squats, crossing, stand ankle movement, backward movement of arms, sit and stand)<br>35-45 min endurance exercise (directional steps, walking)<br>6 min stretching<br><br>Intensity (number of repetitions) began at 65% of Heart Rate reserve, increased to 70% after 3-months.<br><br>Phone contacts were scheduled for the weeks 4, 10 and 22, as well as home visits or in-person meetings for weeks 6 and 18.<br>**Control/comparator**<br>N=15<br>Intervention group 2 (IG2): the same home-based CR program using a paper booklet instead as a guide.<br><br>N=16<br>Control group (CG): education regarding the cardiovascular risk factors. Daily walks were also encouraged.	portion size of food groups (fats, carbohydrates, etc.)	NS<br><br>**Nutrition outcomes 3-months**<br>Total cholesterol (mg/dl) (mean, SD)<br>IG1: 144.6, 59.1<br>IG2: 147.7, 36.1<br>CG: 147.1, 42.5<br>NS<br><br>High-density lipoprotein cholesterol (mg/dl) (mean, SD)<br>IG1: 42.2, 6.3<br>IG2: 40.6, 8.2<br>CG: 43.5, 8.0<br>NS<br><br>Low-density protein cholesterol (mg/dl) (mean, SD)<br>IG1: 78.4, 37.4<br>IG2: 78.9, 18.5<br>CG: 85.3, 38.8<br>NS<br><br>Triglycerides (mg/dl) (mean, SD)<br>IG1: 105.5, 38.6<br>IG2: 124.5, 56.8<br>CG: 92.0, 16.8<br>NS	

Authors<br>***Country***<br>***Years data collected***	***Setting***<br>***Sample characteristics (sample size, demographics)***	***Recruitment method***<br>***Eligibility criteria***	***Study design***<br>***Virtual reality intervention description***<br>***Control/comparators description***<br>***Retention at follow-up/end of treatment***	***Measures of health behaviours***	***Health behaviours outcomes***	***Secondary Outcomes***<br><br>***Use of support received***<br>***Satisfaction/acceptability of support received***<br>***Costs***
		infarction or heart procedures. Additionally, those who had significant and uncompensated visual and auditory deficits, those who were uneducated and/or with no fluency in Portuguese and those who had attended or planned to attend gym or regular physical exercise programs were excluded.	**Retention**<br>72% (33/46) retained at 3-months follow-up.<br>Note: 100% men after dropout		**6-months**<br>Total cholesterol (mg/dl) (mean, SD)<br>IG1: 141.6, 26.5<br>IG2: 175.4, 45.4<br>CG: 168.9, 22.8<br>NS<br><br>High-density lipoprotein cholesterol (mg/dl) (mean, SD)<br>IG1: 45.3, 6.4<br>IG2: 39.7, 6.1<br>CG: 48.6, 10.1<br>NS<br><br>Low-density protein cholesterol (mg/dl) (mean, SD)<br>IG1: 71.4, 28.2<br>IG2: 98.9, 34.4<br>CG: 97.7, 21.5<br>NS<br><br>Triglycerides (mg/dl) (mean, SD)<br>IG1: 104.1, 38.2<br>IG2: 156.0, 65.2<br>CG: 100.6, 14.0<br>S ($p<0.034$)	

Authors Country Years data collected	*Setting Sample characteristics (sample size, demographics)*	*Recruitment method Eligibility criteria*	*Study design Virtual reality intervention description Control/comparators description Retention at follow-up/end of treatment*	*Measures of health behaviours*	*Health behaviours outcomes*	*Secondary Outcomes*<br><br>*Use of support received Satisfaction/acceptability of support received Costs*
**Obesity/Weight, Nutrition, Physical Activity**						
Sullivan et al. [51]<br>USA<br>NA	Kansas City (n=20)<br><br>**Sex**<br>85% (17/20) female<br>**Age (mean, SD)**<br>31.1, 3.6 years<br>**BMI (mean, SD)**<br>32.8, 5.1	NA<br><br>**Eligibility criteria**<br>Overweight or obese individuals, access to high-speed internet, were free from disease as determined by a health history, did not smoke, and were sedentary, defined as <500 kcal/week of exercise.	**Study design**<br>RCT<br><br>3 months of weight loss with a weekly clinic delivered via FTF or VR and then 6 months' weight maintenance delivered with VR.<br>Clinics are 60 minutes in length and use behavioural strategies to promote change in diet and exercise.<br>**Intervention**<br>N=10<br>Second Life only (SLO): Participants in Second Life create virtual representations of themselves, called "avatars," which can interact with other avatars and navigate through the virtual world of Second Life.<br>On the island, the Second Life weight management center contains a conference room for clinic meetings and several areas where learning experiences with real-time feedback were conducted. These included a virtual home with a stocked kitchen, grocery store, restaurant, buffet line, holiday party environment, and gym.<br>Participants communicated with each other and interacted with the health educator through their computer	**Physical activity**<br>Number of steps calculated using pedometer, Self-reported minutes of exercise (300 min/week)<br><br>**Nutrition**<br>Self-reported: Daily number of fruit and vegetable consumption. Consumption of pre-packaged meals.<br><br>**Obesity/weight**<br>Weight (kgs) at 3, 6 and 9-months using a digital scale accurate to ±0.1kg.	**Physical activity outcomes**<br>*Weekly mean minutes of physical activity (min/wk)*<br>*9-months (mean, SD)*<br>FTF: 160, 45<br>SLO: 386, 266<br>NS<br><br>*Pedometer mean number of steps per week (steps/wk)*<br>*9-months (mean, SD)*<br>FTF: 59,903, 12,405<br>SLO: 82,124, 15,433<br>S (P < .05)<br><br>**Nutrition outcomes**<br>9-months (mean, SD)<br>*Mean Daily consumption of fruit*<br>FTF: 1.9, 0.4<br>SLO: 2.7, 0.7<br>S (P<.05)<br>*Daily consumption of vegetables*<br>FTF: 1.8, 0.3<br>SLO: 2.5, 0.7<br>NS (p=0.07)	**Adherence**<br>NS mean difference in clinic attendance between groups.

Authors Country Years data collected	*Setting Sample characteristics (sample size, demographics)*	*Recruitment method Eligibility criteria*	*Study design Virtual reality intervention description Control/comparators description Retention at follow-up/end of treatment*	*Measures of health behaviours*	*Health behaviours outcomes*	*Secondary Outcomes*<br><br>*Use of support received Satisfaction/acceptability of support received Costs*
			keyboard or by using a headset. Participants navigated the area using an interactive map.<br><br>**Control/comparator**<br>N=10<br>Face-to-face (FTF: 10-minute protocol to obtain attendance, weight, and self-reported minutes of physical activity, steps (per step counter), number of fruits and vegetables, and pre-packaged meals consumed.<br>Health educator delivered a Weight Control Research Project (WCRP) lesson for approximately 30 minutes from the topics of nutrition, physical activity, and lifestyle modification.<br>Final 20 minutes of group discussion and problem solving.<br>Experiential learning assignments given to practice successful weight management. A mid-week check-in was conducted via phone, fax or email to report on physical activity and dietary compliance.<br><br>**Retention**<br>NA	Height was measured using a stadiometer BMI was calculated as kilograms per square meter.	**Obesity/weight outcomes**<br>Mean % weight change Baseline to 3 months (mean %, SD)<br>FTF: 10.8%, 3.5%<br>SLO: 7.6%, 5.1%<br>S (p<0.5)<br><br>4-9 months (% increase/decrease)<br>FTF: regained 13.6%<br>SLO: lost 3.7%<br><br>*Adjusted weight change*<br>Significant Group x Period interaction (P < .05).	
